# Kinesin Khc-73/KIF13B modulates retrograde BMP signaling by influencing endosomal dynamics at the *Drosophila* neuromuscular junction

**DOI:** 10.1371/journal.pgen.1007184

**Published:** 2018-01-26

**Authors:** Edward H. Liao, Lindsay Gray, Kazuya Tsurudome, Wassim El-Mounzer, Fatima Elazzouzi, Christopher Baim, Sarah Farzin, Mario R. Calderon, Grant Kauwe, A. Pejmun Haghighi

**Affiliations:** 1 Buck Institute for Research on Aging, Novato, CA, United States of America; 2 Department of Physiology, McGill University, Montreal, QC, Canada; Brandeis University, UNITED STATES

## Abstract

Retrograde signaling is essential for neuronal growth, function and survival; however, we know little about how signaling endosomes might be directed from synaptic terminals onto retrograde axonal pathways. We have identified Khc-73, a plus-end directed microtubule motor protein, as a regulator of sorting of endosomes in *Drosophila* larval motor neurons. The number of synaptic boutons and the amount of neurotransmitter release at the *Khc-73* mutant larval neuromuscular junction (NMJ) are normal, but we find a significant decrease in the number of presynaptic release sites. This defect in *Khc-73* mutant larvae can be genetically enhanced by a partial genetic loss of Bone Morphogenic Protein (BMP) signaling or suppressed by activation of BMP signaling in motoneurons. Consistently, activation of BMP signaling that normally enhances the accumulation of phosphorylated form of BMP transcription factor Mad in the nuclei, can be suppressed by genetic removal of *Khc-73*. Using a number of assays including live imaging in larval motor neurons, we show that loss of Khc-73 curbs the ability of retrograde-bound endosomes to leave the synaptic area and join the retrograde axonal pathway. Our findings identify Khc-73 as a regulator of endosomal traffic at the synapse and modulator of retrograde BMP signaling in motoneurons.

## Author summary

Retrograde axonal transport is essential for normal synaptic function and neuronal survival. How endosomes are specifically sorted from the synaptic terminal for retrograde axonal transport is currently not known. At the *Drosophila* neuromuscular junction, receptors for the Bone Morphogenic Protein signaling pathway are transported from the synapse to the neuron cell body for the proper establishment of synaptic growth and function of motoneurons. Using this system we demonstrate that a kinesin motor protein, *Khc-73*, is necessary for the efficient sorting of retrograde bound vesicles to the retrograde transport route.

## Introduction

Bidirectional communication between the neuronal cell body and distant synaptic terminals is essential for synapse formation, plasticity and neuronal survival [1, 2]. This is achieved primarily through highly regulated axonal transport. Anterograde transport is mediated by plus-end directed kinesin motor proteins that deliver synaptic vesicles and newly synthesized proteins to the synapse, while retrograde transport of cargo destined for the cell body, such as activated receptor complexes, is accomplished by dynein protein complexes [1, 3–5]. Kinesin and dynein motors are also required for endosomal traffic within the cell. The coordinated action of anterograde and retrograde motors ensures the proper sorting and delivery of signaling complexes, proteins and organelles [6]. Although defects in endosomal traffic and axonal transport have been associated with a number of nervous system diseases including Charcot-Marie-Tooth disease, Amyotrophic Lateral Sclerosis, Huntington’s disease and Parkinson’s disease, we know little about how signaling endosomes are routed from the synapse to the retrograde pathway [7–13]. Retrograde signaling has been extensively studied at the *Drosophila* larval neuromuscular junction (NMJ). In particular the Bone Morphogenic Protein signaling pathway (BMP) has been identified as a major regulator of synaptic growth and function. As such, many regulators of synaptic endosomal sorting have been identified in the regulation of BMP signals at synaptic terminals. Nevertheless, how activated receptors are preferentially sorted to travel to the nucleus is currently unknown.

The movement of endosomes within the cytoplasm is directed through the actions of microtubule binding proteins such as minus end dynein motors, and plus end directed kinesins. Co-ordination and competition between these opposing motors for endosome cargoes regulates the transport of proteins to their correct targets [1, 4, 14–18]. In this study, we have discovered a surprising role for the plus-end directed microtubule motor protein Khc-73 in *retrograde* sorting of signaling vesicles at the *Drosophila* larval NMJ.

Khc-73 and its vertebrate homolog KIF13B/GAKIN are kinesin 3 motor protein family members with multiple protein domains and diverse roles in both vertebrates and invertebrates [15, 19–32]. At its N-terminal, Khc-73 contains a kinesin motor necessary for its association with microtubules and plus-end directed transport to synaptic terminals, and at its C-terminal, a Cytoskeletal Associated Protein GLYcine rich (CAP-GLY) domain that provides microtubule association properties [30, 32]. In the nervous system, through microtubule cytoskeleton interactions, both KIF13B and Khc-73 have been shown to participate in mechanisms that control neuronal polarity: Khc-73 has a role in spindle orientation in neuroblasts [30], and KIF13B is involved in the establishment of axonal structures in post-mitotic neurons [20]. Interestingly, KIF13B/Khc-73 has been implicated in the regulation of endosomal dynamics [33, 34] and axonal transport [15] through interaction with Rab5-GTPases. Our previous findings suggested that Khc-73, under strong inhibitory control of the microRNA miR-310-313 cluster in motoneurons at the *Drosophila* NMJ, plays an important role in the regulation of synaptic function by influencing presynaptic neurotransmitter release [35].

In order to investigate the mechanism of action of Khc-73, we have generated loss of function deletions in *Khc-73* gene in *D*. *melanogaster* and examined the motoneurons of third instar larvae. While the number of synaptic boutons at the NMJ and the amount of neurotransmitter release per action potential are unaffected in *Khc-73* mutant larvae, we find a small but significant decrease in the number of presynaptic release sites. Our experiments indicate the presence of Khc-73 function in BMP signaling by demonstrating a strong genetic interaction between *Khc-73* and members of the BMP signaling pathway. We further show that activation of retrograde BMP signaling that normally leads to accumulation of pMad in the nuclei of motoneurons is significantly suppressed when *Khc-73* is genetically removed. Our findings suggest that Khc-73 exerts its function by influencing the sorting of endosomes at the NMJ and promoting retrograde routing of endosomes. Our findings identify, for the first time, a plus-end directed microtubule motor protein as a regulator of retrograde signaling in motoneurons.

## Results

### *Khc-73* loss of function mutant

Khc-73 is a member of the KIF superfamily of kinesin motor proteins and the homologue of the vertebrate KIF13B/GAKIN (S1A Fig) [30, 35]. We have previously shown that *Khc-73* is a target of the micro RNA miR-310-313 cluster in motoneurons. We found that loss of the miR-310-313 cluster led to abnormally enhanced neurotransmitter release at the NMJ; this enhancement was fully reversed to wild type levels as a result of neuronal knock down of Khc-73 [35]. In order to investigate the role of Khc-73 in more detail, we generated deletions in the *Khc-73* gene by imprecise excision of a P-element transposon insert in the vicinity of the 5’ UTR of *Khc-73* (S1B Fig). We isolated two deletion flies *Khc-73*^*149*^ and *Khc-73*^*193*^ missing portions of the *Khc-73* 5’UTR and the ATG start (S1B Fig); we also isolated a fly where the P-element was excised precisely leaving the entire genetic region of *Khc-73* intact (*Khc-73*^*100*^) (S1B Fig). Our western blot analysis with an antibody against the C-terminal end of Khc-73 indicates that both *Khc-73*^*149*^ and *Khc-73*^*193*^ are protein null alleles (S1C Fig). Khc-73 is maternally expressed and is expressed in the embryo [32]. We examined the expression pattern of Khc-73 protein in motoneurons with transgenic overexpression, since we were not able to detect a specific signal using our antibody against Khc-73 in larval preparations. We overexpressed HA-tagged *Khc-73* transgene in motor neurons and detected punctate accumulation of HA-Khc-73 both in axons and in synaptic boutons at the NMJ (S1D and S1E Fig). In addition, we tested transcriptional activity of *Khc-73* by generating a *Khc-73-Gal4* fly (containing 4kb of *Khc-73* genomic sequence driving Gal4 expression, see methods). Crossing this fly to *UAS-mCD8-GFP* transgene led to the expression of GFP in nearly all neurons including motor neurons, suggesting that *Khc-73* transcription is active in all motor neurons in third instar larvae (S1F and S1G Fig). We also found *Khc-73* transcription widely expressed in the brain of adult flies (S1H Fig).

**Fig 1:**
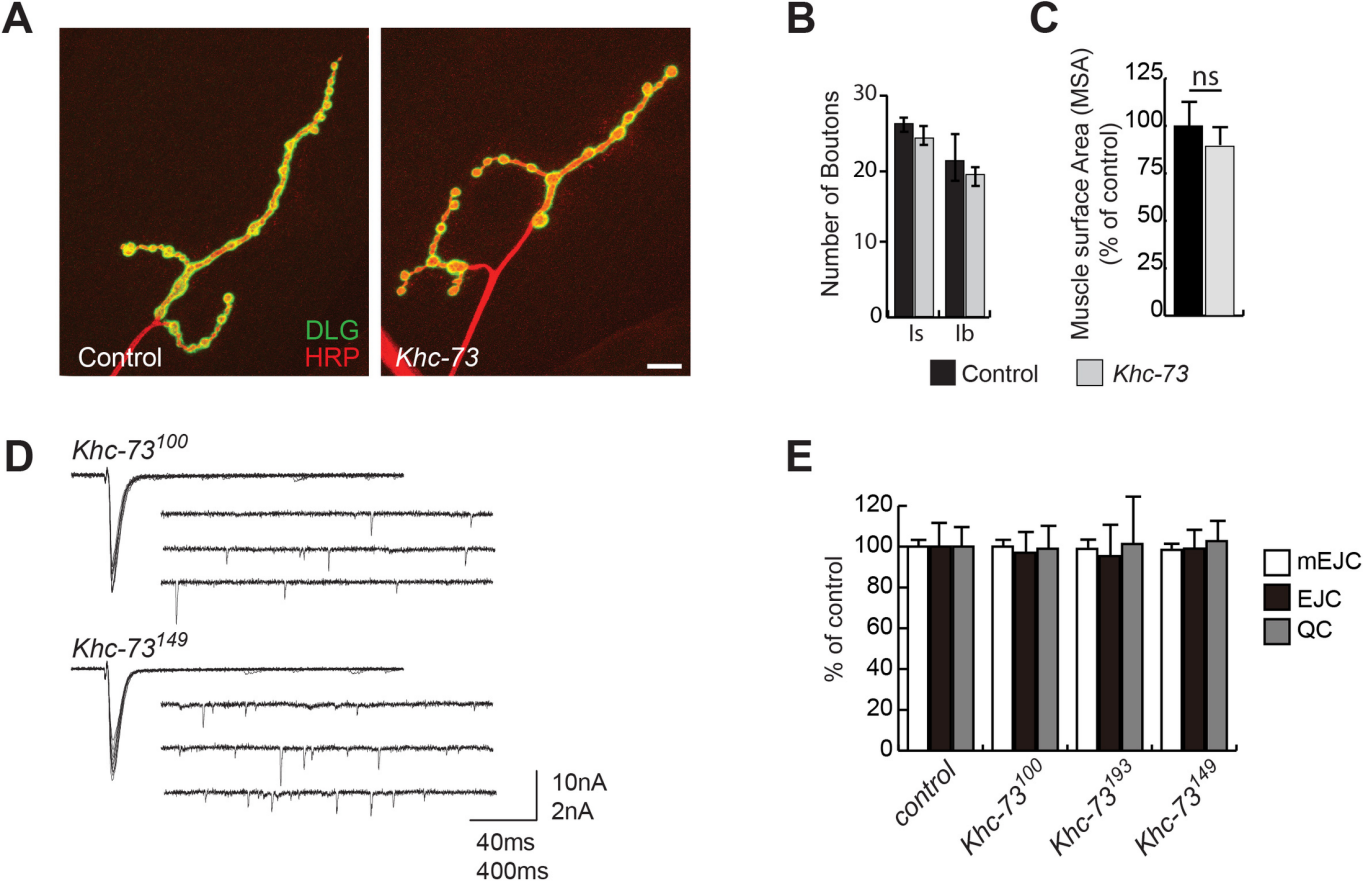
*Khc-73* mutants have normal synaptic structure and function. (A) *Khc-73* synapse structure at muscle 4. Postsynaptic Dlg stain (green) and presynaptic neuron HRP stain (red). Control (*Khc-73*^*100*^). Scale bar is 10μm. (B) Quantification of bouton number in Control (*Khc-73*^*100*^) and *Khc-73* (*Khc-73*^*149*^) mutants at muscle 4 n = 17, 18 NMJs. (C) Muscle surface area of muscle 4 in Control (*Khc-73*^*100*^) and *Khc-73* (*Khc-73*^*149*^). Muscle 4 n = 17, 18. (D) Representative traces of EJCs and mEJCs from third instar larval NMJ in precise excision *Khc-73*^*100*^ (top) and *Khc-73* mutant (*Khc-73*^*149*^) (bottom). (E) Quantification of mEJC, EJC and QC for control *w*^*1118*^, *Khc-73*^*100*^, *Khc-73*^*193*^ and *Khc-73*^*149*^. N = 9, 14, 7 and 11 NMJs. Error Bars are SEM. Student’s t-test. ns-no statistical significance.

Based on the previously published roles for Khc-73 in neuroblasts, endosomal sorting, axon morphology and synaptic function [21, 29, 30, 34–36], we expected loss of *Khc-73* to cause significant defects in the normal synaptic function and/or structure. To our surprise, we found only mild defects (S2E and S2F Fig) in *Khc-73* mutant larvae in our assessment of gross synaptic structure at the larval NMJ. The number of synaptic boutons and the muscle surface area (MSA) at NMJs were not significantly different comparing *Khc-73* mutant and control larvae; this was true for muscle 4 NMJs (Fig 1A–1C) as well as muscle 6/7 NMJs (S1I and S1J Fig).

To test whether loss of Khc-73 might affect synaptic function, we examined the baseline electrophysiological properties including miniature excitatory postsynaptic currents (mEPSCs), evoked excitatory postsynaptic currents (EPSCs) and quantal content (QC) and found no differences between *Khc-73* mutants and wild type larvae (Fig 1D and 1E). Similarly, we tested synaptic vesicle recycling dynamics in *Khc-73* mutant NMJs with high frequency stimulation and found no significant difference in the decay of the synaptic response compared to controls (S1K and S1L Fig). Consistent with the lack of defects in baseline synaptic function, we found no significant changes in the fluorescent intensity of the synaptic vesicle calcium sensor synaptotagmin (SYT), synaptic vesicle marker cysteine string protein (CSP) or synaptic vesicle recycling protein Epidermal growth factor receptor pathway substrate clone 15 (EPS15) in *Khc-73* mutant larvae (S2A–S2D Fig). We found a mild reduction in the staining intensity for the postsynaptic marker Discs large (Dlg) (S2E and S2F Fig) but no differences in the expression level of postsynaptic glutamate receptor subunit A (GluRIIA) (S2G Fig).

Altogether these findings indicate that synaptic growth and function are largely normal in *Khc-73* mutant.

### *Khc-73* mutant larvae have reduced number of presynaptic release sites

**Fig 2:**
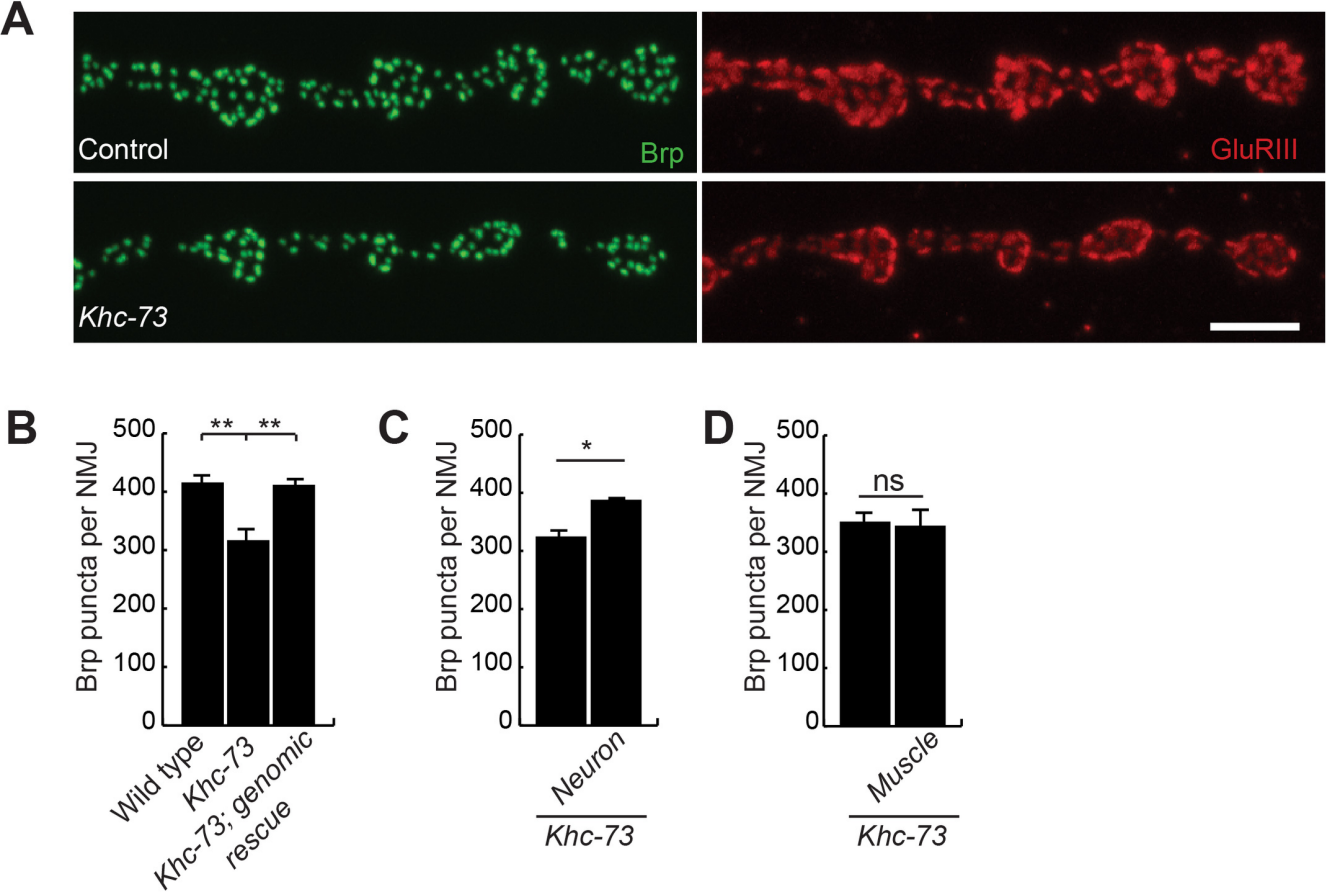
*Khc-73* mutants have fewer active zones. (A) Terminal boutons on muscle 4 NMJs stained with anti-nc82 (Brp) (green) and anti-GluRIII (red) in control (*Khc-73*^*100*^) and *Khc-73*(*Khc-73*^*149*^) third instar larva. Scale Bar is 5μm. (B) Quantification of active zones at muscle 4 NMJ with neuron and muscle rescue. Wild-type (*Khc-73*^*100*^), *Khc-73* (*Khc-73*^*149*^), *Khc-73*; genomic rescue (*Khc-73*^*149*^; *CH321-36I16*/+). N = 36, 19, and 17 NMJs. (C) *Khc-73* neuron rescue of Brp puncta. *Khc-73* control (*OK371-Gal4*, *Khc-73*^*149*^/*Khc-73*^*149*^*)* and Neuron rescue (*OK371-Gal4*, *Khc-73*^*149*^/*Khc-73*^*149*^; *UAS-HA-Khc-73*(*K014*)/+). N = 20, 18 NMJs. (D) *Khc-73* muscle rescue of Brp puncta. *Khc-73* control (*Khc-73*^*149*^; *MHC-Gal4*/+), and *Khc-73* muscle rescue (*Khc-73*^*149*^; *MHC-Gal4*/*UAS-HA-Khc-73*(*K014*)). N = 6 and 6 NMJs. Error bars are SEM. One-Way ANOVA and Student’s t-test. *P<0.05, **P<0.01, ns-no significance.

We have previously reported an increase in the accumulation of the active zone protein Bruchpilot (Brp) in *miR-310-313* cluster mutant larvae that could be reduced by transgenic knockdown of Khc-73 [35]. Therefore, we set out to conduct a deeper examination of *Khc-73* mutants to understand the mechanism of action of Khc-73 in motoneurons. As our previous data would predict, we found a significant decrease in the number of presynaptic release sites per NMJ in *Khc-73* mutant larvae, as indicated by a reduction of the number of Brp Puncta (Fig 2A and 2B). Inclusion of a genomic fragment containing the entire genetic region of *Khc-73* gene restored synaptic defects in *Khc-73* mutant larvae, indicating that this defect is related to loss of *Khc-73* (Fig 2B). Previously we showed that *Khc-73* is under control of the microRNA cluster miR-310-313 [35]. To maintain this relationship in our tissue specific rescue, we used a Khc-73 transgene *Khc-73-3’UTR(K014)* [35] that retains this negative regulatory control. We found that transgenic expression of Khc-73 in presynaptic motoneurons, but not in postsynaptic muscles was sufficient to establish a normal number of presynaptic release sites (Fig 2C and 2D). This result indicates that Khc-73 function in motoneurons is required for normal maturation of synaptic release sites.

### Khc-73 is required for efficient BMP signaling in motor neurons

During larval development, both the coordinated growth of synaptic boutons and the establishment of synaptic strength at the NMJ are largely dependent on a retrograde signaling cascade that is initiated by the release of the Bone morphogenic protein Glass bottom boat (Gbb) in postsynaptic muscles. Gbb signals through type I and type II BMP receptors, leading to phosphorylation of and subsequent accumulation of the BMP transcription factor Mad (Mothers against decapentaplegic) in the nuclei of motor neurons [37–41]. Through this signaling cascade, genes that control synaptic growth and function are transcriptionally regulated [42–44]. The decrease in the number of presynaptic release sites in *Khc-73* mutant larvae, therefore, prompted us to examine the state of BMP signaling in these mutants.

**Fig 3:**
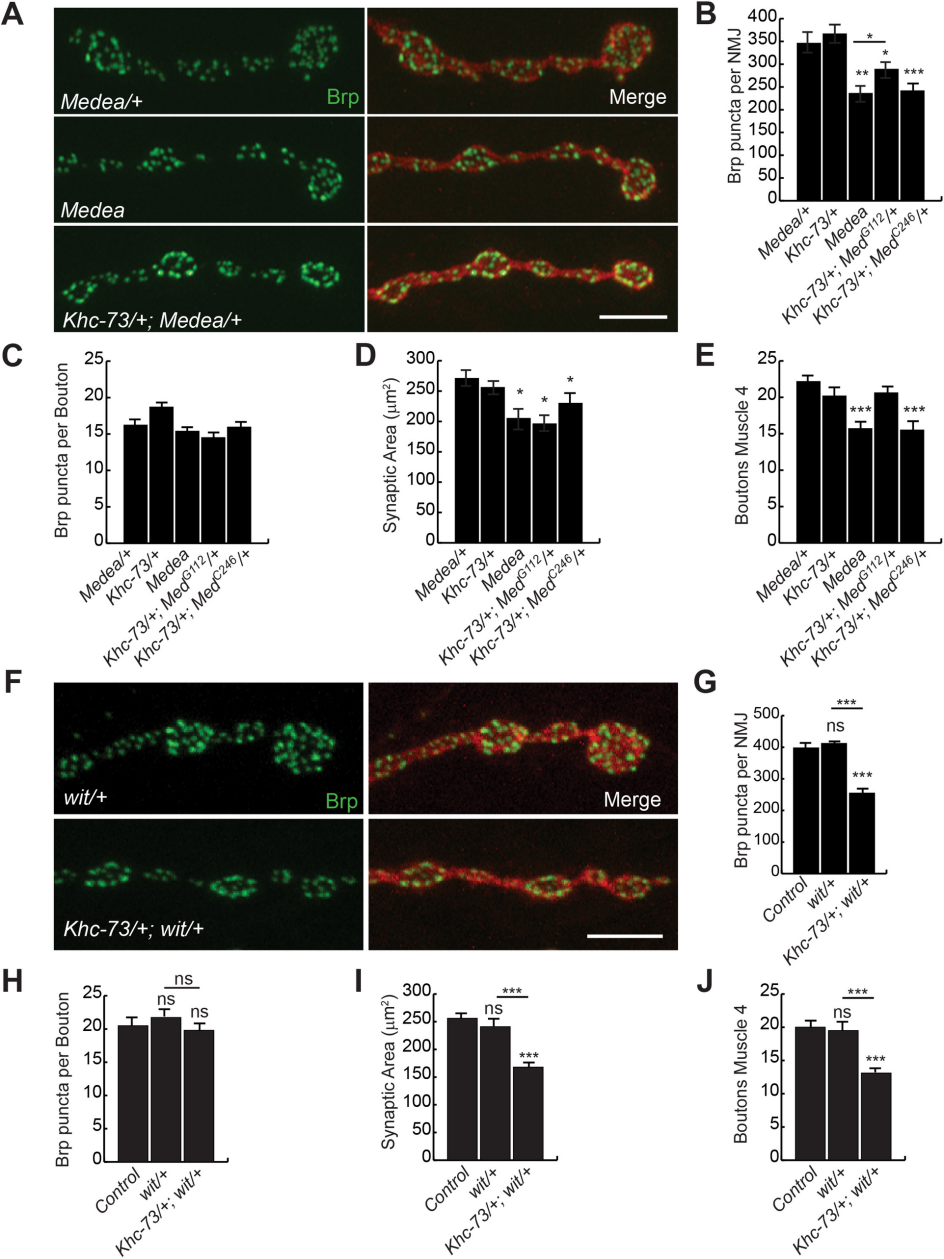
*Khc-73* genetic interaction with BMP pathway. (A) Terminal boutons on muscle 4 of third instar larva in *Medea*/+ (*Med*^*G112*^/+), *Medea* (*Med*^*C246*^/*Med*^*G112*^) and *Khc-73*/+; *Medea*/+ (*Khc-73*^*193*^/+; *Med*^*G112*^/+). Brp (green) and HRP (red). Scale bar is 5μm. (B-E) Quantification of (B) Brp puncta per NMJ, (C) Brp puncta per bouton, (D) HRP synaptic area, (E) Number of boutons at muscle 4 NMJ for *Medea*/+ (*Med*^*G112*^/+), *Khc-73*/+ (*Khc-73*^*149*^/+), *Medea* (*Med*^*C246*^/*Med*^*G112*^), *Khc-73*/+; *Medea*/+ (*Khc-73*^*193*^/+; *Med*^*G112*^/+) and *Khc-73*/+; *Medea*/+ (*Khc-73*^*149*^/+; *Med*^*C246*^/+), N = 31, 17, 17, 21 and 14 NMJs. (F) Terminal boutons on muscle 4 in *wit*/+ (*wit*^*A12*^ /+) and *Khc-73*/+; *wit*/+ (*Khc-73*^*149*^*/+; wit*^*A12*^*/+*) mutant larva. (G-J) Quantification of (G) Brp puncta per NMJ, (H) Brp puncta per bouton, (I) HRP Synaptic area, (J) Number of boutons at muscle 4 NMJs for Control (*Khc-73*^*100*^*/+*), *wit*/+ (*wit*^*A12*^/+) and *Khc-73*/+; *wit*/+ (*Khc-73*^*149*^/+; *wit*^*A12*^/+). N = 11, 8 and 10. Error bars are SEM. One-Way ANOVA and Student’s t-test. *P<0.05, **P<0.01, ***<0.001.

The first indication of Khc-73 involvement with BMP signaling came from genetic interaction experiments between *Khc-73* and the *Drosophila* homolog of vertebrate SMAD4, *Medea*. Medea is a transcriptional co-factor that is required for normal BMP signaling in motor neurons [45]. We found that a combination of previously published alleles *Medea*^*G112*^ and *Medea*^*C246*^ resulted in a very small reduction in the number of boutons at the NMJ compared to *Medea*^*C246*^ homozygous loss of function mutant [45], suggesting that *G112* is a hypomorphic allele (Fig 3A and 3E). Interestingly, in transheterozygous combinations of *Khc-73* and *Medea*, we found a significant reduction in the number of presynaptic release sites per NMJ (Fig 3A and 3B), no change in Brp puncta per bouton (Fig 3C), a significant reduction in synaptic area (Fig 3D) and a significant reduction in bouton number with *Med*^*C246*^ allele but not *Med*^*G112*^ (Fig 3E), as compared to heterozygous *Medea*^*G112*^ controls. This transheterozygous genetic interaction suggested that Khc-73, while having a mild influence on baseline BMP signaling, becomes critical when BMP signaling is compromised. In support of these results, we also found a strong genetic interaction between *Khc-73* mutants and a mutation in the BMP type II receptor *wishful thinking* (*wit*): transheterozygous combination between *Khc-73* and *wit* ^*A12*^ mutants led to a significant reduction in the number of presynaptic release sites (Fig 3F and 3G), synaptic area (Fig 3I) and bouton number (Fig 3J) but no change in Brp puncta per bouton (Fig 3H).

To further explore the functional link between Khc-73 and BMP signaling, we generated double mutant combinations of *Khc-73* and *Medea*. We assessed these double mutant combinations for defects in Brp puncta number at the NMJ and for accumulation of Brp in axons (as previously reported [46]). We found that defects in active zone number and abnormal Brp accumulation in axons in the transallelic combination of *Medea*^*C246*^/*Medea*^*G112*^ were not further enhanced upon removal of *Khc-73* (S3A–S3F Fig), indicating that *Khc-73* and *Med* likely function in the same and not parallel pathways with respect to these phenotypes.

**Fig 4:**
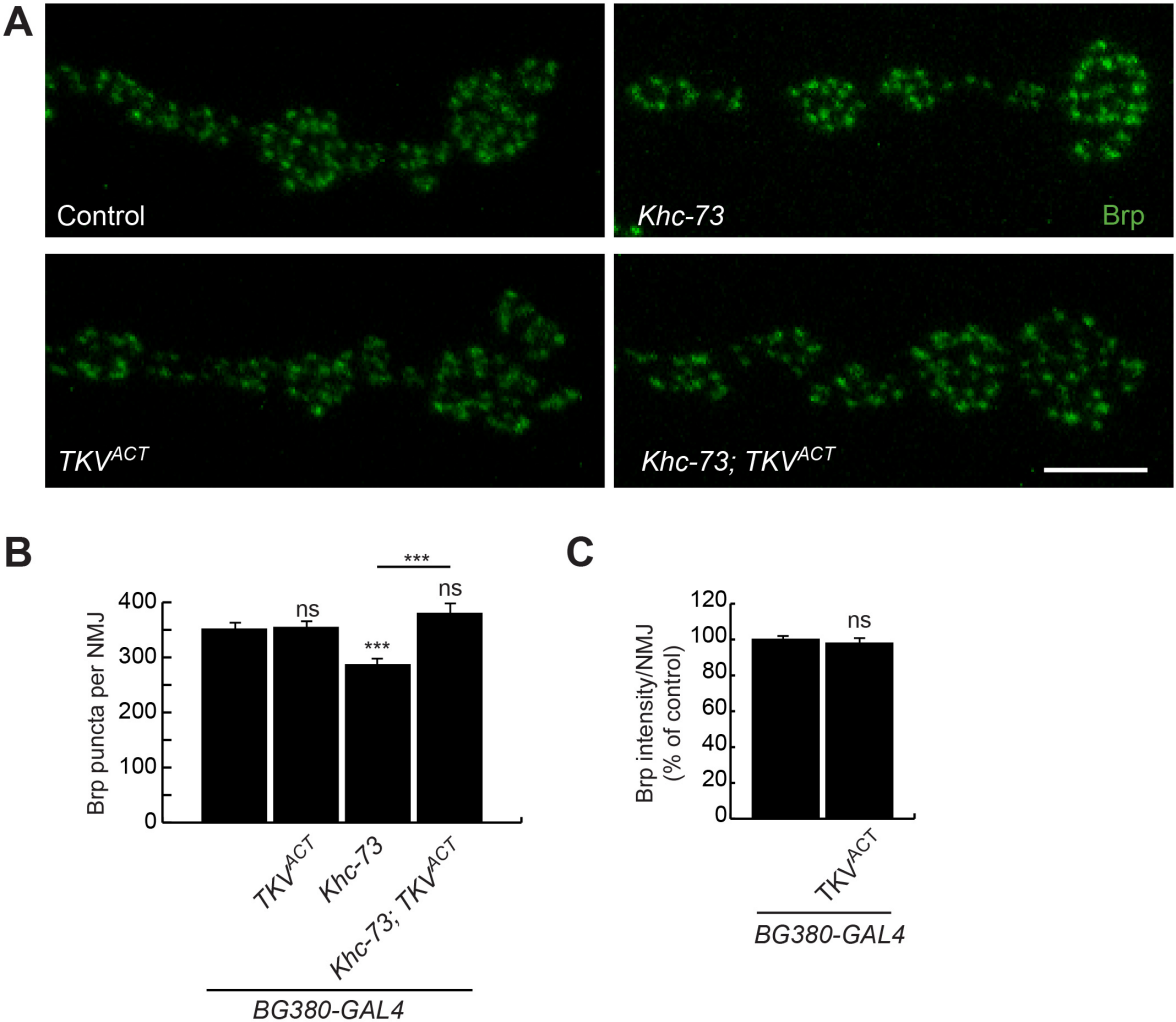
Enhanced BMP signaling suppresses loss of Brp puncta in *Khc-73* mutants. (A) Brp puncta in terminal boutons of muscle 4 NMJs in Control (*BG380-Gal4*/+; *UAS-luciferase*/+), TKV^ACT^ (*BG380-Gal4*/+; *UAS-TKV*^*ACT*^/+), *Khc-73* (*BG380-Gal4*/+; *Khc-73*^*149*^/*Khc-73*^*149*^), *Khc-73*; *TKV*^*ACT*^ (*BG380-Gal4*/+; *Khc-73*^*149*^; *UAS-TKV*^*ACT*^/+) larvae. (B) Quantification of BRP puncta per NMJ in genotypes in (A). N = 18, 17, 13, 12 NMJs. (C) Overexpression of activated form of TKV in motoneurons does not enhance BRP intensity at the NMJ. Control (*BG380-Gal4*/+; *UAS-luciferase*/+); TKV^ACT^ (*BG380-Gal4*/+; UAS-*TKV*^*ACT*^). N = 19, 17 NMJs. Error bars are SEM. Student’s t-test. ***<0.001. ns-no significance. Scale bar is 5μm.

We then tested whether defects in Brp puncta number in *Khc-73* mutants can be restored by overexpressing BMP signaling in motoneurons. Indeed, overexpression of a constitutively active form of BMP type I receptor Thick veins (TKV^ACT^) in motoneurons was capable of reversing the reduction in Brp puncta defect in *Khc-73* mutant larvae (Fig 4A–4C).

**Fig 5:**
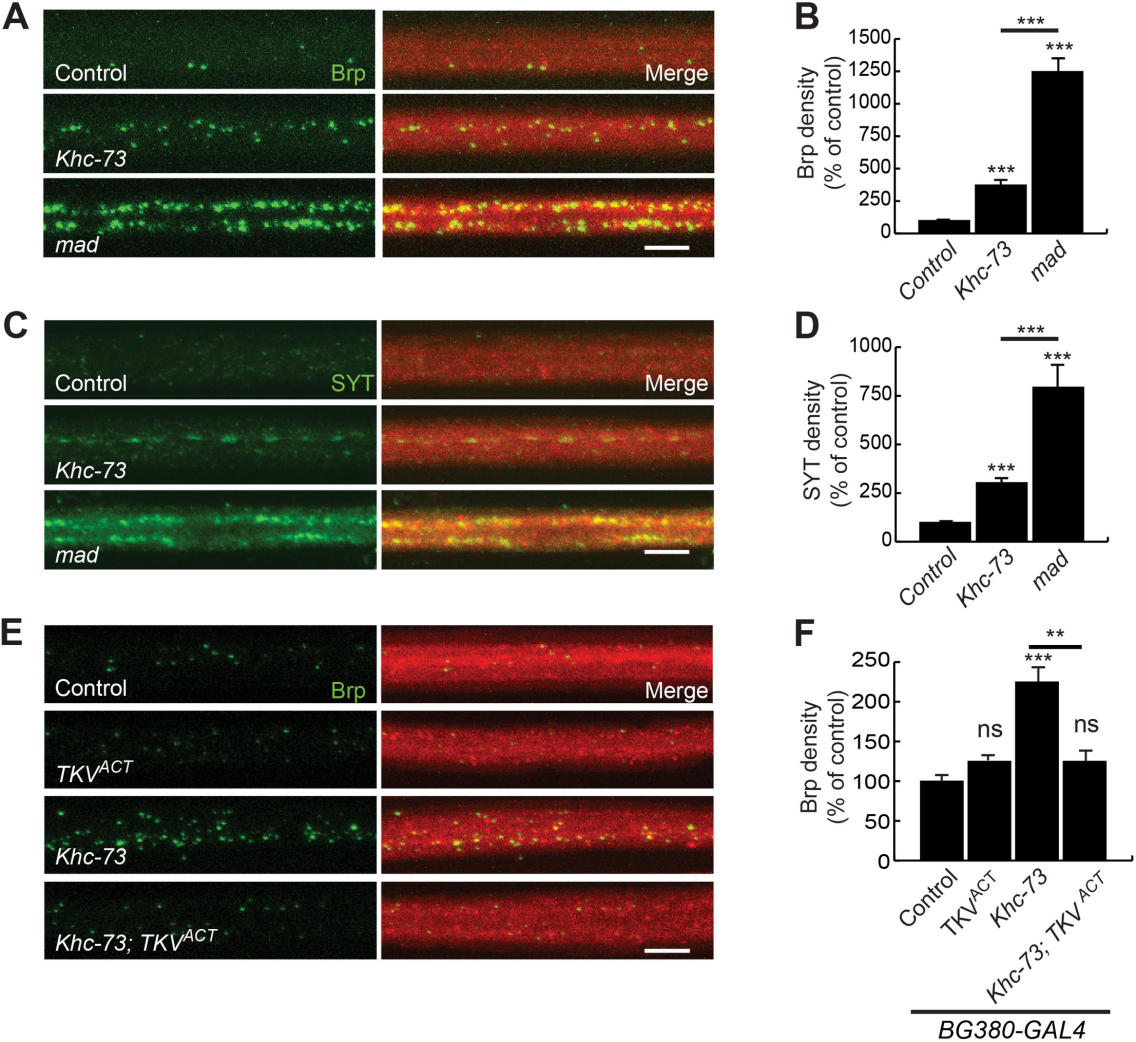
Synaptic proteins Brp and synaptotagmin accumulate in axons of *Khc-73* and *mad* mutants. (A) Brp puncta in axons of Control (*Khc-73*^*100*^*/+*), *Khc-73* (*Khc-73*^*149*^) and *mad* (*mad*^*K00237*^). (B) Quantification of Brp density (Number of Brp puncta/μm^3^) expressed as a percentage of control. N = 19, 10, 10. (C) SYT puncta in axons of Control (*Khc-73*^*100*^*/+*), *Khc-73* (*Khc-73*^*149*^) and *mad* (*mad*^*K00237*^). (D) Quantification of SYT density (Number of Brp puncta/μm^3^) expressed as a percentage of control. N = 19, 10, 10. (E) Brp puncta in axons of Control (*BG380-Gal4*/+), TKV^ACT^ (*BG380-Gal4*/+; *UAS-TKV*^*ACT*^/+), *Khc-73* (*BG380-Gal4*/+; *Khc-73*^*149*^), *Khc-73*; *TKV*^*ACT*^ (*BG380-Gal4*/+; *Khc-73*^*149*^; *UAS-TKV*^*ACT*^/+). (F) Quantification of Brp puncta axon density for genotypes in (E). N = 10, 5, 10 and 9 larvae. Error Bars are SEM. Student’s t-test. **P<0.01, ***P<0.001. ns-no statistical significance. Scale bar is 5μm.

These results prompted us to compare the degree of axonal accumulation of Brp and another synaptic marker, synaptotagmin (SYT) between *Khc-73* and *Mad* mutants. For this we used a *Mad* mutant allele (*Mad*^*K00237*^) that is known to show a strong reduction of synaptic growth and function at the NMJ and exhibit defects in axonal transport of synaptic markers [41]. Both endogenous Brp and SYT accumulated in large aggregates in axons of *Mad* mutant larvae compared to wild type or *Khc-73* mutant larvae, highlighting the fact that *Khc-73* related axonal defects would be comparable to a hypomorphic loss of function of BMP signaling (Fig 5A–5D). In support of this interpretation, the increase in Brp accumulation in axons of *Khc-73* mutant larvae was fully reversed as a result of overexpression of TKV^ACT^ (Fig 5E and 5F).

We also tested whether abnormal axonal accumulation of Brp in *Khc-73* mutant larvae could be due to changes in microtubule structures; however, we found no significant changes in the expression of acetylated tubulin in axons or terminals in *Khc-73* mutant larvae when compared to wild type counterparts (S3G and S3H Fig). These results further support that the defects associated with active zone numbers in *Khc-73* mutant larvae are most likely related to defects in BMP signaling.

**Fig 6:**
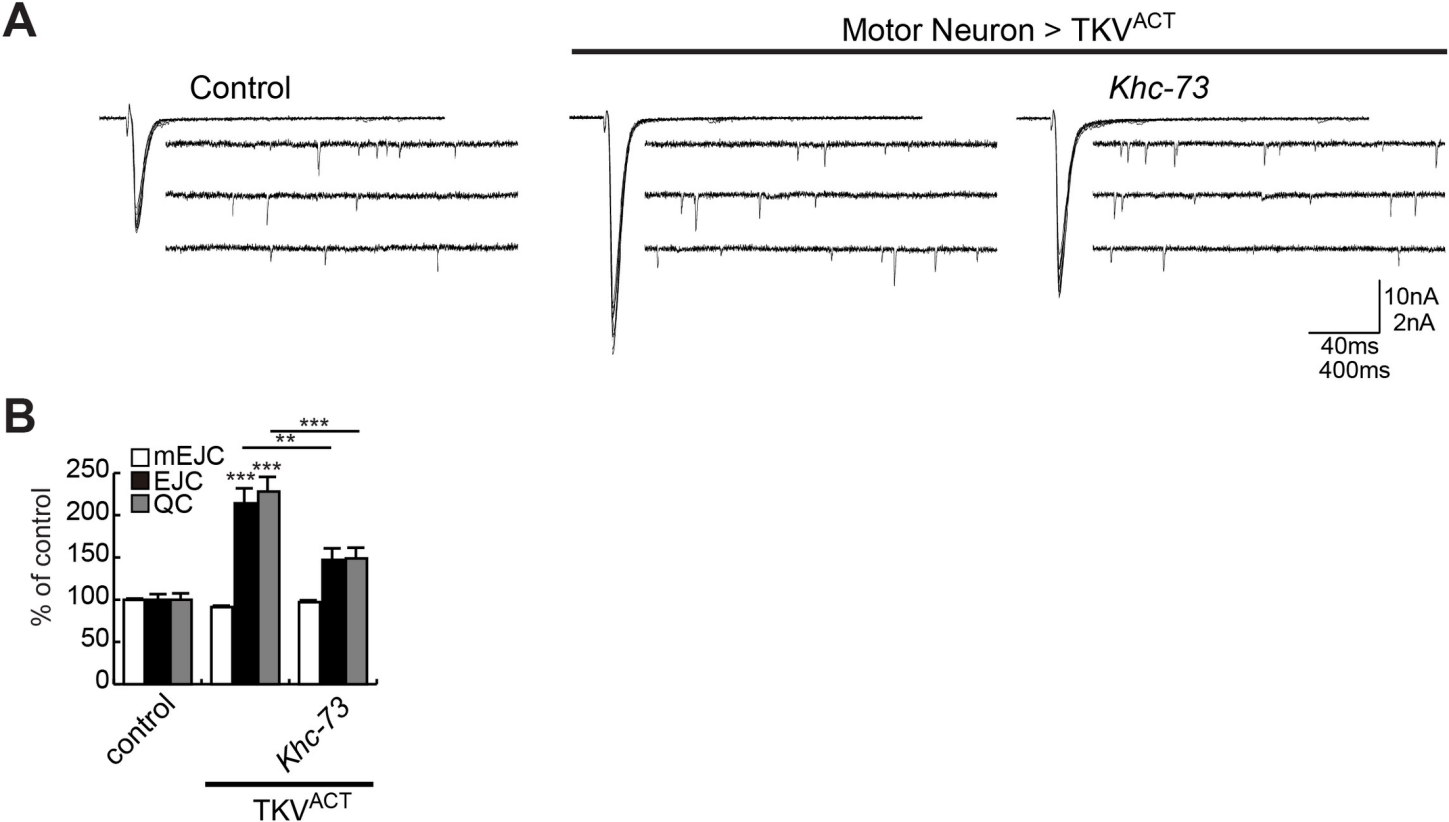
*Khc-73* suppression of BMP pathway activation at the NMJ. (A) Representative traces of EJC and mEJCs of control (*BG380-Gal4*/+), Motor Neuron>TKV^ACT^ (*BG380-Gal4*/+; *UAS-TKV*^*ACT*^/+) and Motor Neuron>TKV^ACT^, *Khc-73* (*BG380-Gal4*/+; *Khc-73*^*149*^; *UAS-TKV*^*ACT*^/+). (B) Quantification of mEJC, EJC and QC of genotypes shown in (A). n = 18, 18 and 20. Error bars are SEM. One-Way ANOVA and Student’s t-test. **P<0.01, ***<0.001.

While we did not detect measurable changes in the accumulation of pMad in response to loss of *Khc-73* (S4A and S4B Fig), or overexpression of *Khc-73* transgene (S4C and S4D Fig), the genetic interactions described above provide strong evidence for a functional link between Khc-73 and BMP signaling. In order to strengthen this link and extend it to the regulation of synaptic function, we conducted a number of electrophysiological examinations. Mild to moderate overexpression of TKV^ACT^ in motor neurons can lead to an enhancement in synaptic release without significantly affecting the number of synaptic boutons [44, 47]. We found that loss of *Khc-73* could significantly block the ability of TKV^ACT^ to enhance synaptic strength (Fig 6A and 6B).

**Fig 7:**
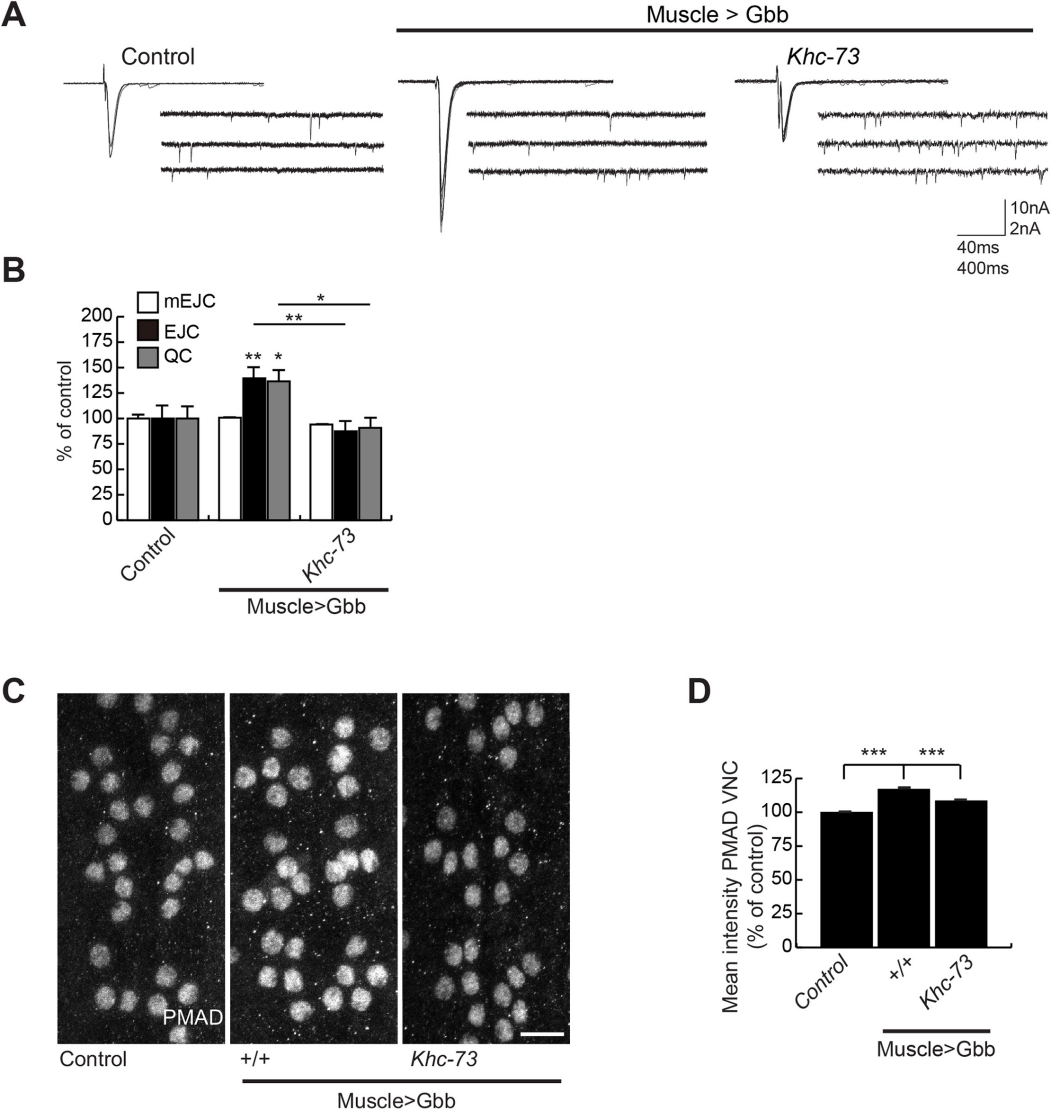
Khc-73 is required for retrograde BMP signaling at the NMJ. (A) Representative traces of EJC and mEJCs of Control (*MHC-Gal4*/+), Muscle>Gbb (*UAS-Gbb*^*99*^/+; *MHC-Gal4*/+) and Muscle>Gbb, *Khc-73* (*Khc-73*^*193*^, *UAS-Gbb*^*99*^/*Khc-73*^*149*^, +; *MHC-Gal4*/+). (B) Quantification of mEJC, EJC and QC for genotypes shown in (A). n = 10, 10 and 7. (C) pMad staining in the motoneuron nuclei of ventral nerve cord in MHC Control (*MHC-GAL4*/+), MHC>Gbb (*UAS-Gbb*^*99*^/+; *MHC-GAL4*/+) and MHC>Gbb, *Khc-73* (*Khc-73*^*193*^, *UAS-Gbb*^*99*^/*Khc-73*^*149*^, +; *MHC-GAL4*/+) larvae. (D) Quantification of the mean fluorescence intensity of nuclei for genotypes in (C). n = 302(7), 372(9) and 5(210), Nuclei (larvae) respectively. Error Bars are SEM. One way ANOVA. Student’s t-test. *P<0.05, **P<0.01, ***P<0.001.

Similarly, we found that overexpression of the BMP ligand Gbb in postsynaptic muscles led to a significant enhancement in quantal content (Fig 7A and 7B). As in the case of TKV^ACT^ induced enhancement in neurotransmitter release, loss of *Khc-73* led to a significant suppression of Gbb-induced enhancement in neurotransmitter release (Fig 7A and 7B).

In addition, we quantified the accumulation of pMad in motoneuron nuclei in the ventral nerve cord (VNC) as a result of postsynaptic overexpression of Gbb. It is well accepted that the accumulation of pMad in the nuclei of motor neurons is a reliable readout of the strength and efficiency of retrograde BMP signaling in motor neurons and is essential for BMP-dependent transcriptional regulation as well as regulation of synaptic function [38, 40, 44, 48, 49]. Muscle overexpression of Gbb led to a statistically significant increase in pMad in the nuclei of motoneurons, which was fully reversed as a result of loss of *Khc-73* (Fig 7C and 7D).

**Fig 8:**
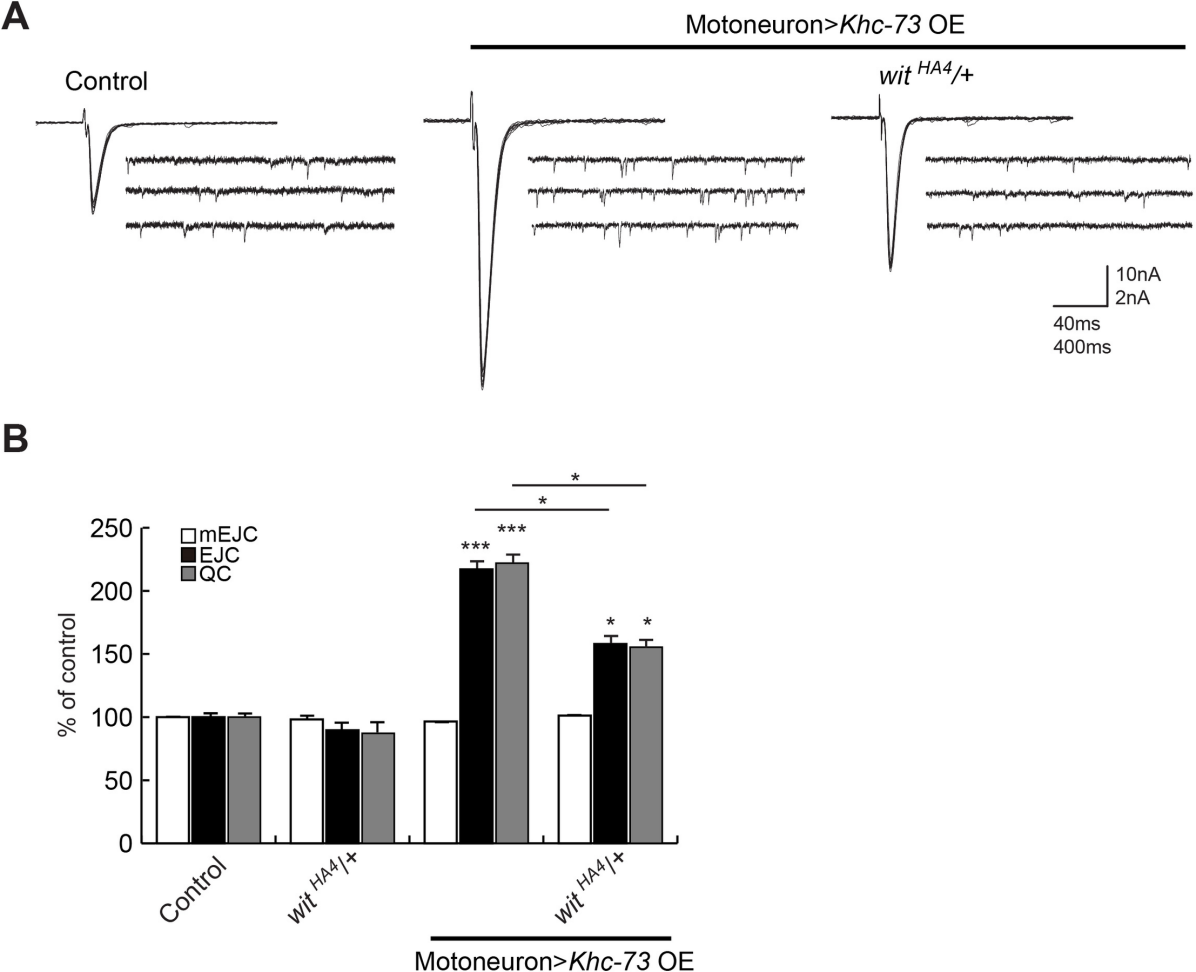
Khc-73 induced enhancement of synaptic release is suppressed by heterozygosity in BMP receptor *wit*. (A) Representative traces for EJCs and mEJCs in control (*OK371-Gal4*/+; *UAS-luciferase*/+), Khc-73 OE (*OK371-Gal4*/*UAS-Khc-73*) and Khc-73 OE; *wit*^*HA4*^/+ (*OK371-Gal4*/*UAS-Khc-73*; *wit*^*HA4*^/+). (B) Quantification of mEJC, EJC and QC for genotypes in control (*OK371-Gal4*/+; *UAS-luciferase*/+), *wit*^*HA4*^/+ (*OK371-Gal4*/+; *wit*^*HA4*^/+), Khc-73 OE (*OK371-Gal4*/*UAS-Khc-73*) and Khc-73 OE; *wit*^*HA4*^/+ (*OK371-Gal4*/*UAS-Khc-73*; *wit*^*HA4*^/+). N = 10, 10, 10 and 10. Error Bars are SEM. Student’s t-test. *P<0.05, **P<0.01, ***P<0.001. ns-no statistical significance.

Finally, we tested whether Khc-73 gain-of-function would be dependent on normal BMP signaling in motoneurons. We have previously shown that Khc-73 overexpression in motoneurons leads to an enhancement of neurotransmitter release [35]. We found that heterozygosity for the BMP type II receptor *wishful thinking* (*wit*) was sufficient to suppress this enhancement to a large extent (Fig 8A and 8B), further supporting the presence of a functional link between Khc-73 and BMP signaling.

From these results a picture emerges, indicating a strong functional link between Khc-73 and BMP signaling in motor neurons. But how does Khc-73 interact with BMP signaling? BMP signaling in motoneurons depends on tightly regulated endosomal traffic. For example, pMad accumulation in motoneuron nuclei in response to activation of BMP signaling at the synapse is dependent on retrograde routing of signaling endosomes containing BMP receptor complexes from the nerve terminal along axons to the cell body [5]. Conversely, routing of BMP receptor complexes to lysosomal pathways appears as one of the mechanisms that attenuates BMP signaling in motor neurons [50–52]. Therefore, we considered a role for Khc-73 in both retrograde routing as well as lysosomal sorting of BMP receptor complexes.

**Fig 9:**
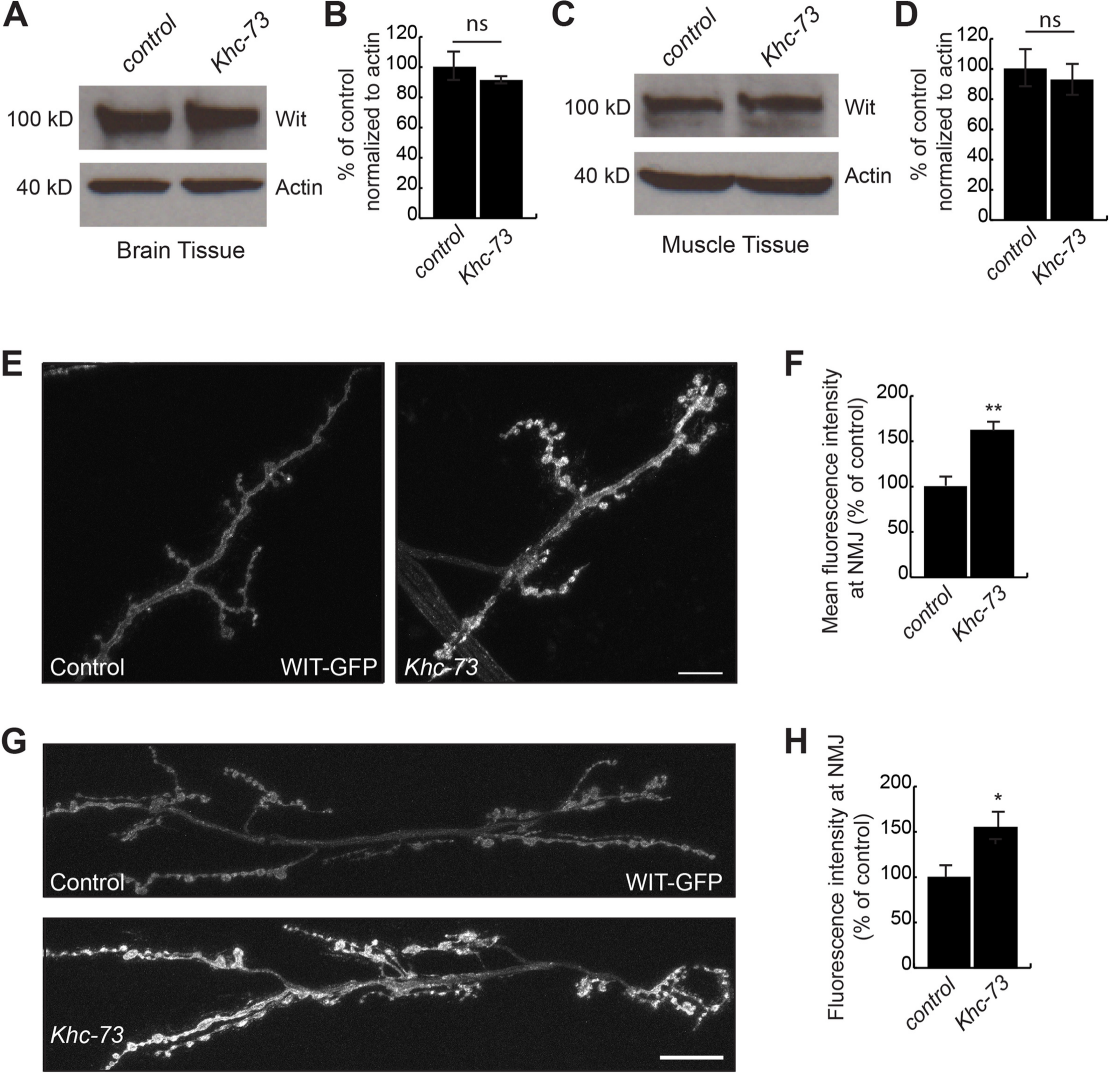
BMP receptors Wit and TKV accumulate at *Khc-73* mutant NMJs. (A) Representative western blot of Wit levels in brain tissue from control (*w*^*1118*^) and *Khc-73* (*Khc-73*^*149*^) mutants. Actin loading control. (B) Quantification of Wit protein band intensity normalized to actin of (A) and shown as percentage of control. n = 3 blots. (C) Representative western blot of Wit levels in muscle tissue from control (*w*^*1118*^) and *Khc-73* (*Khc-73*^*149*^) mutants. Actin loading control. (D) Quantification of Wit protein band intensity normalized to actin for (C) and shown as percentage of control. n = 3 blots. (E) Live image of muscle 4 NMJs in unfixed larvae for Control (*BG380-Gal4*/+; *OK371-Gal4*/ *UAS-Wit-GFP*) and *Khc-73* (*BG380-Gal4*/+; *Khc-73*^*149*^, *OK371-Gal4* / *Khc-73*^*149*^, *UAS-Wit-GFP*). Scale bar is 10μm. (F) Quantification of mean fluorescence intensity as percentage of control for genotypes in (E). N = 11, 6. (G) Live image of muscle 6/7 NMJs in unfixed larvae for Control (*BG380-Gal4*/+; *OK371-Gal4*/*UAS-Wit-GFP*) and *Khc-73* (*BG380-Gal4*/+; *Khc-73*^*149*^, *OK371-Gal4*/ *Khc-73*^*149*^, *UAS-Wit-GFP*). Scale bar is 20μm. (H) Quantification of mean fluorescence intensity as percentage of control for genotypes in (G). N = 10, 9 NMJs. Error Bars are SEM. Student’s t-test. *P<0.05, **P<0.01. ns-no statistical significance.

To test these possibilities, we assessed the level of BMP receptors Wit and TKV using a combination of Western blot analysis and immunohistochemistry. Western blot analysis of CNS and body wall muscle tissue (containing NMJ terminals) revealed no change in the level of endogenous Wit protein as a result of genetic removal of *Khc-73* (Fig 9A–9D). The available antibody to Wit does not detect endogenous Wit in immunohistochemistry. Thus we turned to transgenic Wit and Tkv to visualize their localization at the synapse. Static images of the boutons in live preps of WIT-GFP revealed punctate accumulations at the NMJ and an increase of Wit receptor intensity in *Khc-73* mutants at muscle 4 and muscles 6/7 (Fig 9E–9H). Similarly, TKV:YFP transgene expression appeared more punctate at muscle 4 (S5A Fig) and muscles 6/7 (S5C Fig), trending towards increased intensity at muscle 4 (S5B Fig), while significantly increasing in intensity at muscles 6/7 (S5D Fig) in *Khc-73* mutants. We ruled out changes in TKV:YFP transgene transcription by quantitative PCR (S5E Fig) and did not observe obvious changes in axonal traffic of TKV:YFP in motoneurons (S5F Fig).

**Fig 10:**
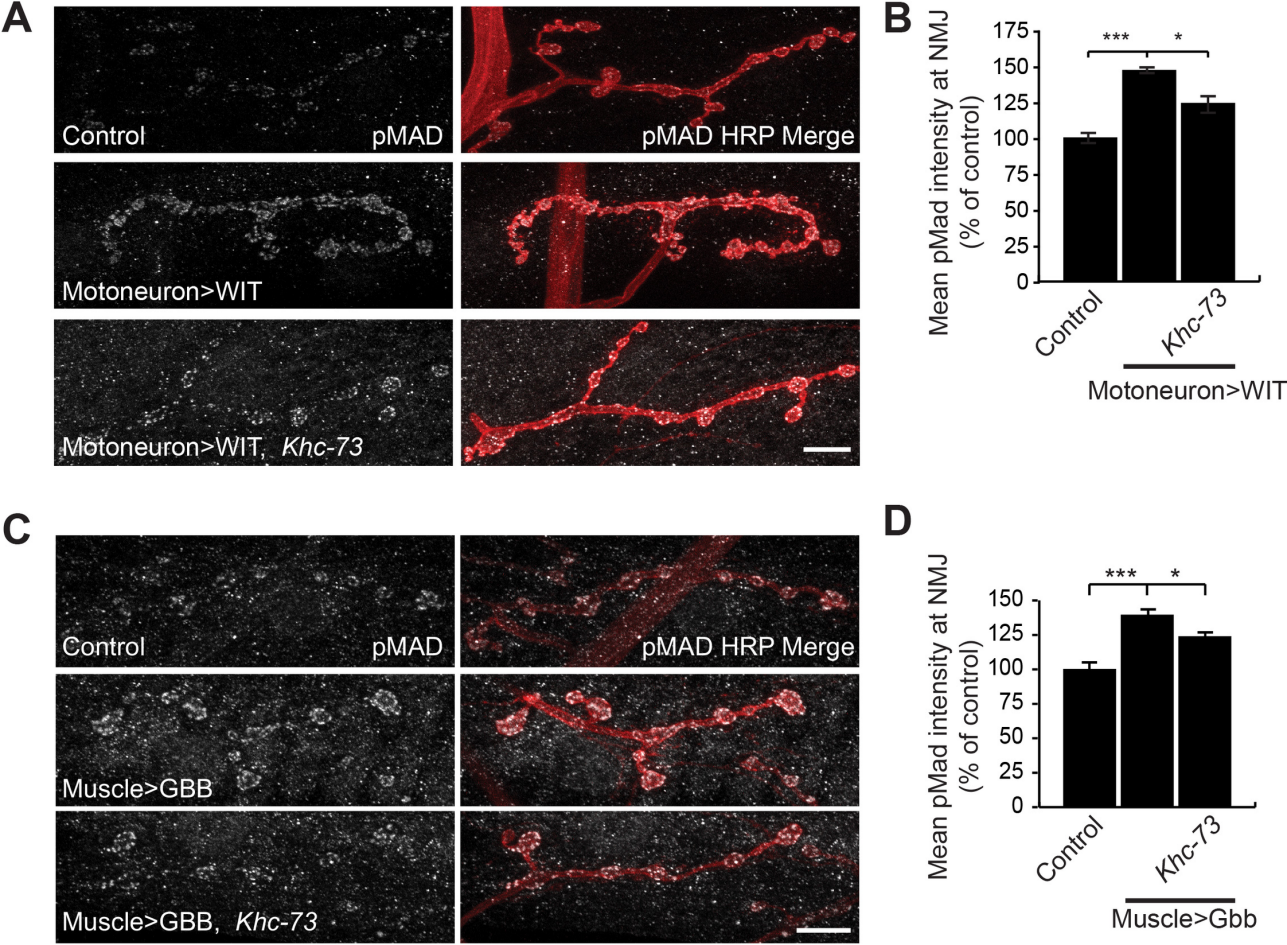
Activation of BMP signaling is suppressed in *Khc-73* mutant larvae. (A) pMad staining at the NMJ for Control (*BG380-GAL4*/+), Motoneuron>WIT (*BG380-GAL4*/+; *UAS-Wit*/+) and Motoneuron>Wit, *Khc-73* (*BG380-GAL4*/+; *UAS-Wit*, *Khc-73*^*149*^/+, *Khc-73*^*149*^). (B) Quantification of mean pMad fluorescence intensity for genotypes in (A). N = 22, 44 and 10 NMJs. (C) pMad staining at the NMJ for Control (*MHC-Gal4*/+), Muscle>Gbb (*UAS-Gbb*^*99*^/+; *MHC-Gal4*/+) and Muscle>Gbb, *Khc-73* (*Khc-73*^*193*^, *UAS-Gbb*^*99*^/*Khc-73*^*149*^, +; *MHC-Gal4*/+) (D) Quantification of mean pMad fluorescence intensity for genotypes in (C). N = 12, 12, and 15 NMJs. Error bars are S.E.M. Student’s t-test. *P<0.05, ***P<0.001. Scale bar is 10μm.

We next tested our model that Khc-73 loss can suppress BMP signaling by examining pMAD levels in larvae overexpressing the Wit receptor in presynaptic neurons and in larvae overexpressing the Gbb ligand from postsynaptic muscle. Overexpression of Wit enhanced presynaptic pMad levels (Fig 10A and 10B). In *Khc-73* mutants, this enhancement was significantly suppressed (Fig 10A and 10B). Similarly, muscle overexpression of Gbb enhanced pMAD levels in presynaptic boutons. *Khc-73* loss also suppressed this increase (Fig 10C and 10D).

It has been demonstrated that BMP receptor activity can be dampened when trapped inside the lumen of multivesicular bodies (MVBs) at the NMJ [53]. Generally, MVBs are intracellular vesicles that contain one or more smaller vesicles within their lumen and play an important role in signal transduction and endosomal sorting [54, 55]. Current evidence suggests that MVBs may be at the crossroads for endosomal cargo joining the lysosomal pathway, the retrograde pathway or the exosomal secretory pathway [55, 56]. We find that fluorescence intensity of the MVB localized protein Hrs (hepatocyte growth factor related tyrosine kinase substrate) is increased by 20% at the NMJ in *Khc-73* mutant larvae overexpressing the BMP receptor Wit (S6A and S6B Fig). Suggesting that there are more MVBs in *Khc-73* mutants in this Wit overexpressing background. Therefore, a scenario can be considered in which retrograde bound BMP receptors are encapsulated in multivesicular bodies and may be stalled at the NMJ in *Khc-73* mutants.

Together, these results suggest that degradation of BMP receptors is not a likely explanation for the inhibition of BMP signaling in *Khc-73* mutant larvae. Secondly, our findings suggest that while BMP receptors appear to accumulate at the NMJs in *Khc-73* mutants, they are in an endosomal state that prevents these receptors from signaling.

### Khc-73 is necessary for proper endosomal sorting at synaptic terminals

**Fig 11:**
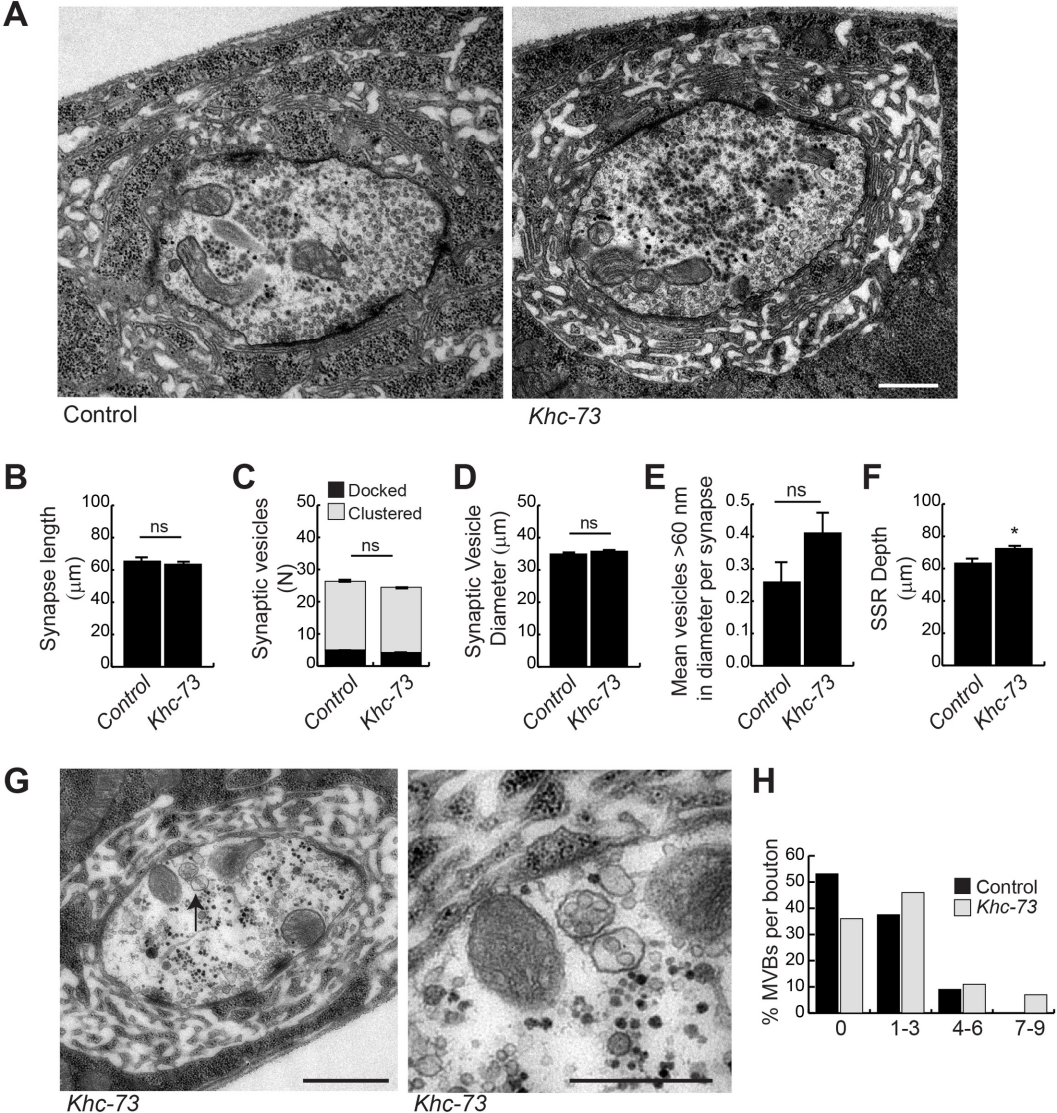
EM analysis of *Khc-73* mutant larvae. (A) EM micrographs of Control (*Khc-73*^*100*^) and *Khc-73* (*Khc-73*^*193*^). Magnification 25000x. Scale bar is 0.5 μm. (B) Quantification of synapse length in Control (*Khc-73*^*100*^) and *Khc-73* (*Khc-73*^*193*^). N = 79, 153 synapses. (C) Quantification of docked and clustered synaptic vesicles at each synapse for Control (*Khc-73*^*100*^) and *Khc-73* (*Khc-73*^*193*^). N = 79, 153 synapses. (D) Quantification of synaptic vesicle diameter for Control (*Khc-73*^*100*^) and *Khc-73* (*Khc-73*^*193*^). N = 2082, 3750 synaptic vesicles. (E) Mean number of vesicles >60nm in diameter at the synapse. N = 79, 153 synapses. (F) Quantification of subsynaptic reticulum (SSR) depth at the synapse. N = 79, 153 synapses. (G) EM micrographs of multivesicular bodies in *Khc-73* mutant. Magnification of MVB denoted by arrow in left panel (right panel). Scale bar 1 μm. (H) Proportion of boutons with indicated number of multivesicular bodies for control (*Khc-73*^*100*^) and *Khc-73* (*Khc-73*^*193*^). N = 33, 28 boutons. Error bars are S.E.M. Student’s t-test. *P<0.05, ns-no statistical significance.

**Fig 12:**
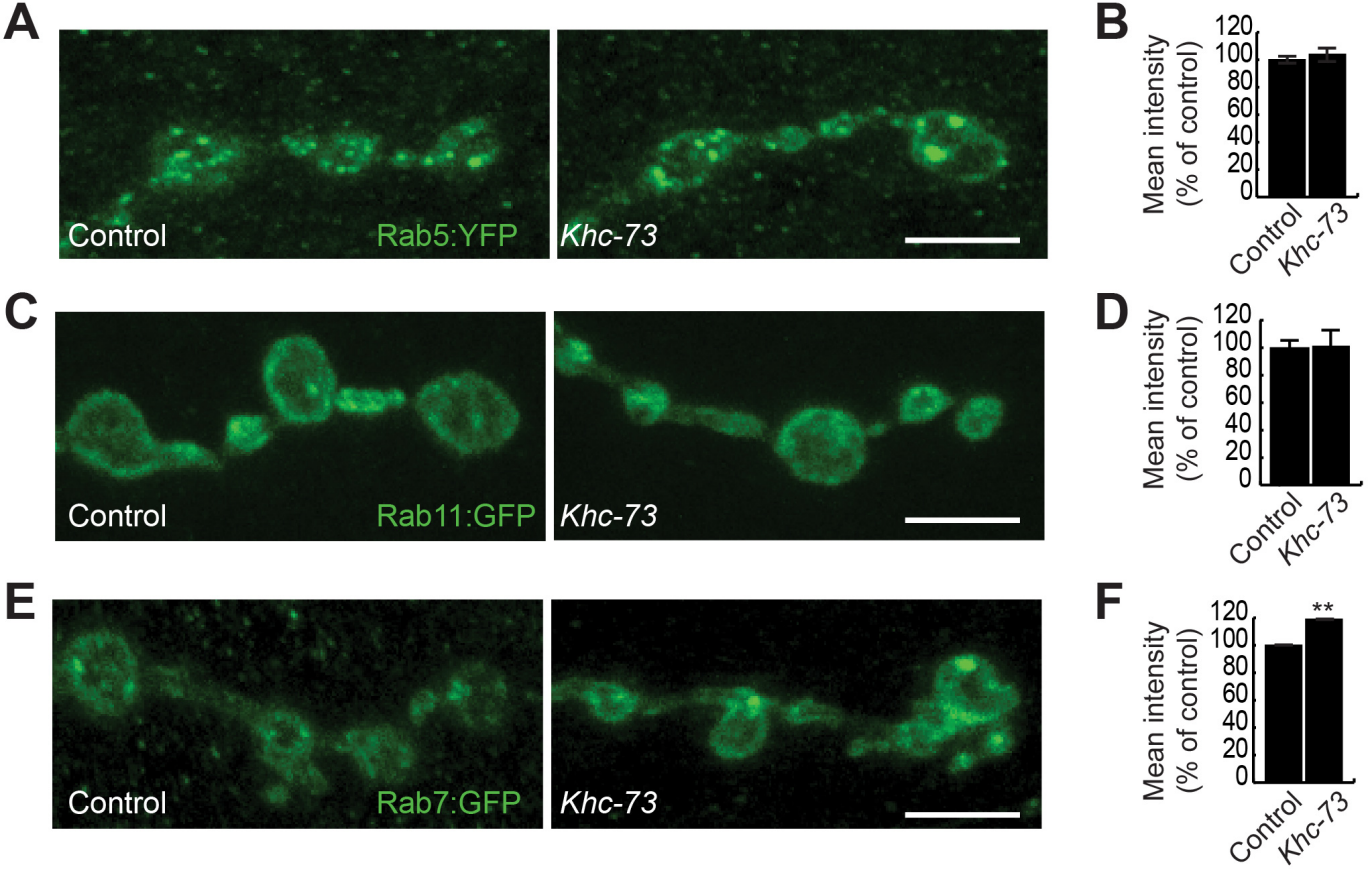
Endosomal markers in *Khc-73* mutant larvae. (A) RAB5:YFP at muscle 4 NMJ terminal boutons stained with anti-GFP in control (*VGlut-Gal4*/+; *UAS-RAB5*:*YFP/+*) and *Khc-73* (*VGlut-Gal4/+; Khc-73*^*149*^/*Khc-73*^*149*^; *UAS-RAB5*:*YFP*). (B) Quantification of RAB5:YFP fluorescence intensity at muscle 4 NMJ for genotypes in (A). n = 8, 12 NMJs. (C) RAB11:GFP at muscle 4 NMJ terminal boutons stained with anti-GFP in control (*OK371-GAL4/ UAS-RAB11*:*GFP*) and *Khc-73* (*OK371-GAL4*, *Khc-73*^*149*^/*Khc-73*^*149*^; *UAS-RAB11*:*GFP*). (D) Quantification of RAB11:GFP puncta fluorescence intensity at muscle 4 NMJs for genotypes in (C). N = 10, 10 NMJs. (E) RAB7:GFP at muscle 4 NMJ terminal boutons stained with anti-GFP in control (*OK371-GAL4/+; UAS-RAB7*:*GFP/+*) and *Khc-73* (*OK371-GAL4*, *Khc-73*^*149*^/+, *Khc-73*^*149*^; *UAS-RAB7*:*GFP*/+). (F) Quantification of RAB7:GFP puncta fluorescence intensity at muscle 4 NMJs for genotypes in (E). N = 20, 17 NMJs. Scale bar is 5μm. Error Bars are SEM. Student’s t-test. **P<0.01.

Previous studies on Khc-73/KIF13B have identified endosomal sorting roles for this protein [15, 21, 22, 27, 28, 34, 57]. In order to gain additional insight into the role of Khc-73 in the regulation of endosomal traffic, we conducted an ultrastructural analysis of NMJ synapses in *Khc-73* mutant larva. Our analysis revealed no gross abnormalities in presynaptic boutons (Fig 11A–11F): different morphometric measures of active zones and synaptic vesicles appeared normal in *Khc-73* mutant larvae (Fig 11B–11E); however, we did detect a small increase in the depth of the subsynaptic reticulum (SSR) (Fig 11F). Interestingly, although we find no statistical difference in the mean MVBs per bouton (1.28±0.29 control and 1.68±0.41 *Khc-73*), we found a proportion of boutons with an abnormally higher number of MVBs (7–9 MVBs per bouton) in *Khc-73* mutant larvae (Fig 11G and 11H). The trend towards more MVBs in *Khc-73* mutant boutons suggested a role for Khc-73 in endosomal sorting. Therefore, we turned to exploring a possible role for Khc-73 in the regulation of endosomal dynamics by examining the expression of transgenic Rab-GTPases at the synapse. Rab-GTPases are small GTPases that associate with endocytic vesicles and are known to mediate many aspects of endosomal traffic in all eukaryotes [58]. Based on previous reports on interaction between Khc-73 with the early endosome associated Rab5 in vitro [34], we tested the expression pattern of Rab5 at the NMJ in *Khc-73* mutant larvae with a *UAS-Rab5*:*YFP* transgene. However, we found that in *Khc-73* mutants the punctate appearance of Rab5:YFP was unaffected in terms of fluorescence intensity or localization (Fig 12A and 12B). Similarly, we did not detect any effect on the expression level of the recycling endosomal marker Rab11 (Fig 12C and 12D). In most eukaryotic cells Rab5 positive internalized vesicles become associated with Rab7 along their path of maturation [59–62]; Rab7 containing late endosomes are then either routed to the lysosomal pathway or the recycling pathway [58, 63]. In neurons, the transition from Rab5 to Rab7 is also necessary for routing late endosomes onto the retrograde pathway [64]. The retrograde pathway is necessary for transporting signaling complexes, neurotrophic factors and other cellular proteins from nerve endings to the cell body [2]. Interestingly unlike the case of Rab5, we found an abnormal increase in Rab7 accumulation at synaptic boutons in *Khc-73* mutants (Fig 12E and 12F). These results suggested to us that the normal dynamics of Rab7 positive vesicles and by extension those of BMP receptors are disrupted in *Khc-73* mutant larvae.

**Fig 13:**
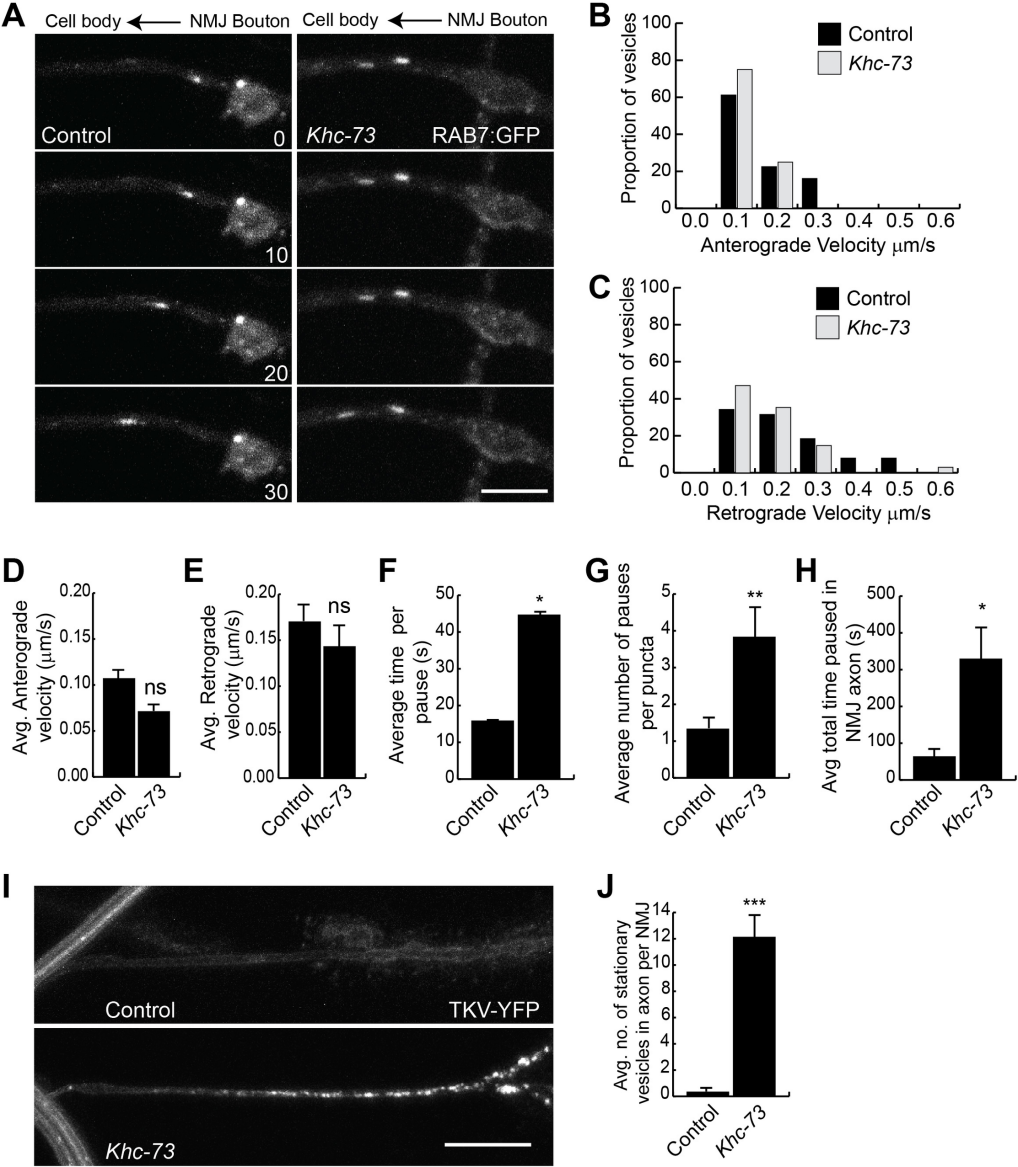
Live imaging of Rab7:GFP at synaptic terminals. (A) Montage of RAB7:GFP retrograde movement at muscle 4 NMJ in control (*OK371-GAL4*/+; *UAS-RAB7*:*GFP*/+) and *Khc-73* (*OK371-GAL4*, *Khc-73*^*149*^/+, *Khc-73*^*149*^; *UAS-RAB7*:*GFP*/+) larvae. Scale bar is 5μm. Time is seconds. (B) Histogram of Anterograde velocities of RAB7:GFP puncta in (A). N = 18(42), 12(36) NMJs(puncta). (C) Histogram of Retrograde velocities of RAB7:GFP puncta in (A). N = 18(42), 12(36) NMJs(puncta). (D) Average anterograde velocity of RAB7:GFP puncta for genotypes in (A). N = 18(42), 12(36) NMJs(puncta). (E) Average retrograde velocity of RAB7:GFP puncta for genotypes in (A). N = 18(42), 12(36) NMJs(puncta). (F) Average time RAB7:GFP puncta spent in each pause event. For genotypes in (A). N = 18(42), 12(36) NMJs(puncta). (G) Average number of pauses per RAB7:GFP puncta.for genotypes in (A). N = 18(42), 12(36) NMJs(puncta). (H) Total time RAB7:GFP puncta remained paused within proximal axon for genotypes in (A). N = 18(42), 12(36) NMJs(puncta). (I) TKV-YFP expression in control (BG380-Gal4/+; UAS-*TKV-YFP*/+) and *Khc-73* (*BG380-GAL4*/+; *Khc-73*^*149*^; *UAS-TKV-YFP*/+) larvae in the NMJ axon of muscle 4. (J) Quantification of the number of stationary puncta observed within the axon from time lapse movies of genotypes in (I). N = 6, 8 NMJs. Scale bar is 5 μm. Error Bars are SEM. Student’s t-test. *P<0.05, **P<0.01, ***P<0.001. ns-no statistical significance.

In order to examine the dynamics of late endosomal traffic in more detail, we set out to conduct live imaging in dissected larvae expressing Rab7:GFP. To see if our observations of Rab7:GFP would be relevant to the dynamics of Wit/Tkv complexes, we confirmed in fixed samples that Wit and Rab7:GFP colocalized when expressed simultaneously (S7A Fig, Pearson’s r coefficient 0.68). We also confirmed that Tkv and Wit colocalized at the NMJ (S7B Fig, Pearson’s r coefficient 0.60). In live dissected larval preparations, Rab7:GFP showed dynamic movement within synaptic boutons in both wild type and *Khc-73* mutants (Fig 13A–13C and S1 Movie and S2 Movie). We noticed that occasionally a Rab7 marked vesicle left the synaptic area and moved retrograde towards the shaft of the axon. Vesicles entering the axon moved, paused and continued moving out of the NMJ. We measured the velocity of these vesicles when in motion and calculated the mean velocity in the anterograde and retrograde directions (Fig 13B–13E, S8A–S8D Fig and S3 Movie and S4 Movie) and found no statistical difference in their velocities. We also recorded the time spent paused in a single spot (Fig 13F), the number of pauses for each spot (Fig 13G) and summed the total time paused in the axon (Fig 13H). Here, our assessment of Rab7 dynamics revealed a significant difference between control and *Khc-73* mutant larvae. We recorded long periods of pausing or stalling of Rab7 positive vesicles in *Khc-73* mutants, which showed statistical difference compared to our recordings in control larvae (Fig 13F and 13H, S1 Movie and S2 Movie). This pausing phenotype provides one explanation for the increase in Rab7:GFP in *Khc-73* NMJs, however alternative explanations related to Rab7:GFP protein turnover are also possible.

We next performed time lapse imaging on TKV-YFP expressing *Khc-73* mutant larvae focusing on the axon shaft near the synapse. Here we observed a similar stalling phenotype of TKV-YFP puncta in *Khc-73* mutants whereas in control larvae the axonal shaft was devoid of stalled puncta (Fig 13I and 13J, S5 Movie and S6 Movie)).

As an additional test for axonal retrograde transport, we used a peripheral axon injury model developed by Collins and colleagues for activating Jun-N-terminal kinase (JNK) signaling in motor neurons [65]. In this model, crushing peripheral axons in larvae leads to a strong transcriptional upregulation of the JNK phosphatase *puckered* (*puc*) in the injured motoneurons [65]. The *puc* transcriptional response to axon injury is dependent on axonal retrograde transport [65]. Using a *puc-LacZ* transcriptional reporter line, we assessed JNK activation in motoneurons in response to nerve crush. In *Khc-73* larvae, we found that *puc* transcriptional upregulation as a result of axonal injury was indistinguishable from that of control larvae (S8E–S8H Fig). Thus we can rule out a defect in retrograde axonal transport in *Khc-73* mutants. Similarly, we did not find any significant changes in axonal transport of mitochondria in *Khc-73* mutant larvae (S7 Movie and S8 Movie). These results provided strong evidence for a model in which Khc-73 is required primarily in synaptic terminals for efficient routing of retrograde vesicles onto the retrograde path with little influence on bidirectional axonal transport.

## Discussion

### Khc-73 is a novel regulator of synaptic endosomal sorting and a modulator of BMP signaling in motoneurons

Khc-73 function plays a supporting role in retrograde BMP signaling under basal conditions. However under conditions of enhanced BMP signaling, this endosomal coordination by Khc-73 becomes critical to transmit the retrograde signal from the synapse to the neuronal cell body.

**Fig 14:**
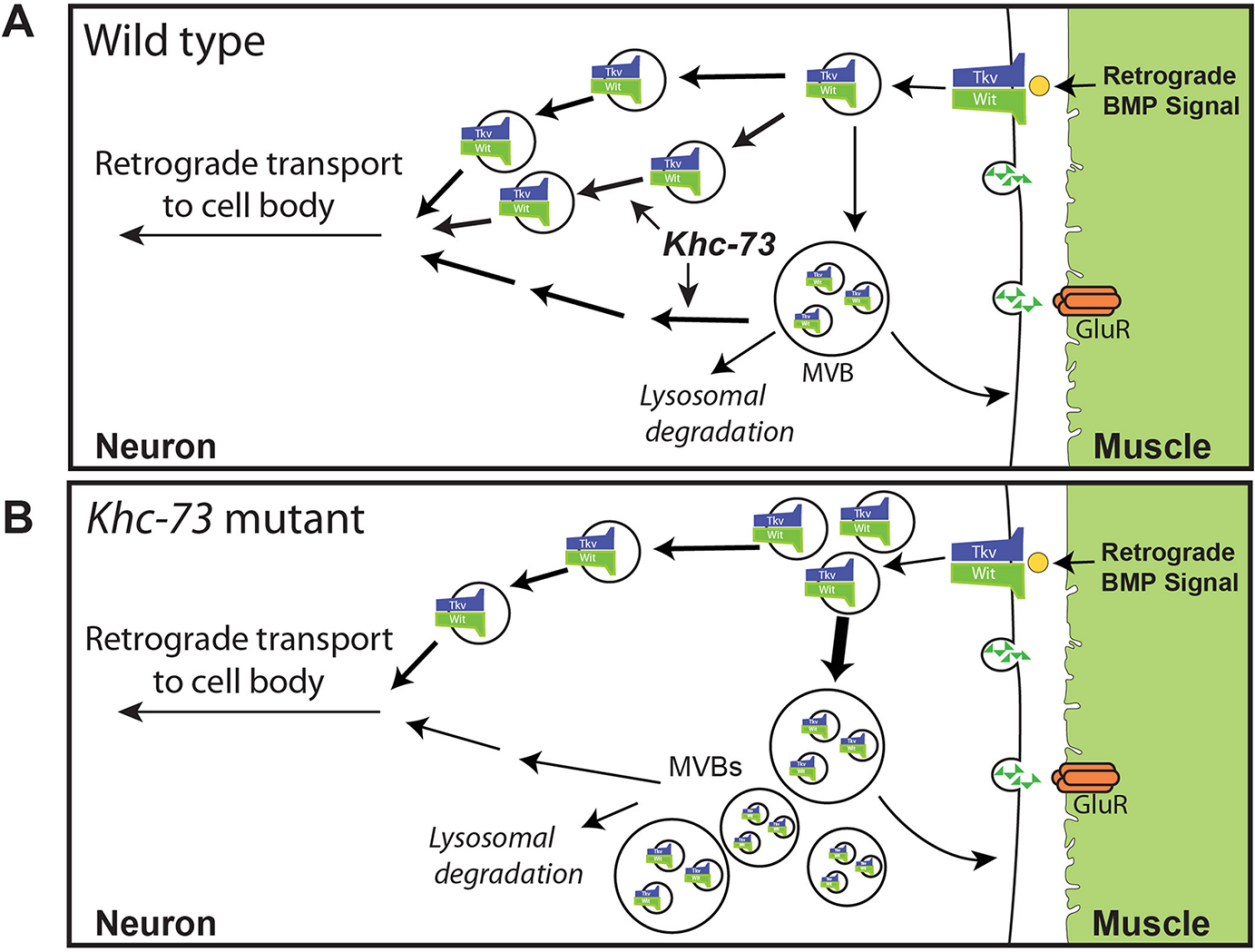
Model slide for Khc-73 function at the synaptic terminal. (A) In wild-type neuromuscular junctions, Khc-73 mediates the efficient transit of retrograde bound endosomes to the retrograde transport machinery. Its function could include enhancement of sorting to retrograde pathways and/or exit of endosomes from the MVB. (B) In the absence of Khc-73, retrograde bound endosomes do not efficiently engage with the retrograde machinery and accumulate in late endosome compartments. This inefficient level of routing is sufficient for basal levels of BMP retrograde signaling. However, when BMP signaling is enhanced, Khc-73 function is needed to mediate the exit of retrograde endosomes from the synapse. In its absence, retrograde bound signaling complexes are slowed in their exit from the synapse, they accumulate and signaling to the cell body is inhibited.

Efficient retrograde signaling from synaptic terminals back to the neuronal soma is critical for appropriate neuronal function and survival [2, 7–11]. Nevertheless, we know very little about the molecular steps that facilitate the routing of synaptic endosomes destined for retrograde axonal pathways. Here we describe several lines of evidence for a potential role for Khc-73 in this process. *Khc-73* mutant larvae develop grossly normal synaptic structure and function at the *Drosophila* larval neuromuscular junction (NMJ), but we find a reduction in the number of presynaptic release sites. Through genetic interaction experiments, we show that this defect is most likely the result of abnormal BMP signaling in motoneurons: transheterozygous combinations of *Khc-73* and *Medea* or *wit* mutants show a significant loss of presynaptic release sites compared to control. Khc-73 becomes even more critical, when higher demand is put on the motoneuron by activating BMP signaling: loss of Khc-73 largely blocks the retrograde enhancement in synaptic release in response to activation of BMP pathway in motor neurons. Consistently we have previously shown that transgenic knock down of Khc-73 in motoneurons blocks the ability of the NMJ to undergo retrograde synaptic homeostatic compensation [35]. Our findings show that when BMP signaling is activated, loss of Khc-73 reduces the accumulation of pMad in motoneuron nuclei, suggesting a role for Khc-73 in the regulation of retrograde signaling. Our immunohistochemical assessment and live imaging analysis of *Khc-73* mutant larvae provide evidence for involvement of Khc-73 in at least two steps in endosomal dynamics in motoneurons. On the one hand, Khc-73 is required for normal dynamics of internalized endosomes through late endosomal and multivesicular stages, and on the other Khc-73 plays a role in facilitating the routing of endosomes onto the retrograde pathway (see Fig 14A for model). These defects have two main consequences: first, we find an accumulation of BMP receptors at the NMJ (possibly in multivesicular bodies) without increased local signaling, suggesting that these receptor containing endosomes might be trapped in a state between late endosomal and lysosomal stage (see Fig 14B for model). Second, we see a dampening of the ability of retrograde bound Rab7:GFP tagged endosomes to join the retrograde pathway, illustrating a defect in retrograde movement of vesicles and possibly providing an underlying explanation for the reduction in pMAD when retrograde BMP signaling is activated in *Khc-73* mutants. These results together present Khc-73, a plus-end microtubule motor, in the unexpected role of regulation of endosomal traffic from synapse to the soma in motoneurons with a role for ensuring the efficiency of retrograde BMP signaling.

While our findings provide compelling evidence for the proposed model above, we cannot, at this time, rule out the possibility that the abnormal accumulation of BMP receptors at the NMJ and the slowing of retrograde movement of Rab7 positive endosome in *Khc-73* mutant larvae could be due to a defect in an intermediate molecule, whose anterograde transport is dependent on Khc-73. In support of such model, we do report an abnormal accumulation of Brp and SYT (two synaptic proteins) in axons. While our data suggests that this abnormal accumulation can be remedied by transgenic activation of BMP signaling in *Khc-73* mutants, we cannot rule out the possibility that an anterograde transport defect might exist for other proteins independent of the interaction between Khc-73 and BMP signaling.

### Khc-73 interactions with dynein, microtubules and endosomes

Our findings point to a model in which Khc-73 facilitates the routing of retrograde bound vesicles onto the retrograde axonal pathway. This model predicts coordination between endosomes, dynein motors and kinesin Khc-73. The coordinated involvement of dynein and kinesin motor proteins in the transport and sorting of endosomes has been previously proposed and examples supporting this model are mounting [14, 66, 67]. Previously published data for Khc-73 and KIF13B have provided evidence that interaction between early endosomes, dynein motors and microtubules are possible. Khc-73/KIF13B is capable of binding to the GTPase Rab5 (found on early endosomes), thus allowing Khc-73 to localize directly to Rab5 endosomes [15, 34, 68]. As a kinesin motor protein, Khc-73 could then transport these endosomes to the retrograde pathway by moving along the microtubule network in the synapse.

Compelling evidence for a dynein interaction with Khc-73 has been previously demonstrated during mitotic spindle formation [24]. The Khc-73/KIF13B stalk domain is phosphorylated by Par1b and this creates a 14-3-3 adapter protein binding motif [29]. It has been proposed that physical interaction between Khc-73 stalk domain and the dynein interacting protein NudE via 14-3-3 ε/ζ might underlie the interaction between Khc-73 and dynein that is necessary for appropriate mitotic spindle formation [24]. Interestingly, transgenic knock down of NudE in *Drosophila* larval motoneurons leads to a reduction in the number of presynaptic release sites, a phenotype reminiscent of *Khc-73* loss of function [69]. Thus, Khc-73 contains domains and protein-protein interactions that are capable of coordinating endosomes, microtubules and dynein. We propose that Khc-73 is necessary for the normal endosomal sorting and exit of endosomes from the NMJ to support efficient retrograde BMP signaling.

## Methods

### Fly stocks

Flies were cultured at 25°C on standard medium except for Gene Switch experiments where RU486 was added to the media (50μM). The following stocks were used: *Med*^*C246*^ (Y324term mutation)[45] and *Med*^*G112*^ (mutation in splice donor site of exon 4)[45] from Herman Aberle [45]. *wit*^*A12*^ [37, 39, 70]. *wit*^*HA4*^ [37, 45, 49]. *UAS-TKV*^*ACT*^ and *UAS-Gbb*^*99*^ [40] provided by M.B. O’Connor (University of Minnesota, Minneapolis, MN), *UAS-Wit* [39], *UAS-HA-Khc-73* and *UAS-HA-Khc-73-3’UTR(K014)* [35], *UAS-Wit-GFP*, *UAS-TKV-YFP* [5], *BG380-Gal4* [71], *Elav-Gal4* [72], *OK371-Gal4*[73], *MHC-Gal4* [74]. Bloomington stocks used were P{y[+m8] = Mae-UAS.6.11}*Khc-73*[DP00530] (RRID:BDSC_22058), *UAS-Rab5*:*YFP* (RRID:BDSC_9775), *UAS-Rab7*:*GFP*(RRID:BDSC_42706), *UAS-Rab11*:*GFP* (RRID:BDSC_50782), *VGlut-Gal4* (RRID:BDSC_24635), *Mad*^*K00237*^ (RRID:BDSC_10474), *UAS-Mito-HA-GFP* (RRID:BDSC_8442), *nSyb-Gal4* (RRID:BDSC_51635). *UAS-luciferase* (RRID:BDSC_35788). *puckered LacZ* insertion *puc*^*E69*^ [75]. Wild type stock used was *w*^*1118*^.

### Deletion of *Khc-73* by P-element excision

*Khc-73* deletions were created by mobilizing the P-element from *y*[1] *w*[67c23]; P{y[+m8] = Mae-UAS.6.11}*Khc-73*[DP00530]. Virgin *y*[1] *w*[67c23]; P{y[+m8] = Mae-UAS.6.11}*Khc-73*[DP00530] female flies were mated to *Cyo*/+; *Δ2–3*, *Sb*/*TM6b* males. Male progenies of *y*[1] *w*[67c23]; P{y[+m8] = Mae-UAS.6.11}*Khc-73*[DP00530]/*Cyo*; *Δ2–3*, *Sb*/+ were mated to virgin *y*, *w*; *CyoGFP*/*Adv* females. Yellow, non Sb, yellow eyed progeny were singly mated to *y*, *w*; *Adv*/*CyoGFP* virgins and individual stocks were established. P-element excisions were screened with the following primers: OED91: CTGACGGCGCTGTTGCTTG and OED96: GATCTAGAGATGATTCTGCATCACTAG TAAAAATT.

### DNA plasmids

*Khc-73* promoter GAL4 construct was generated by cloning a 4kb fragment upstream of the translational start site with primers OED453: CAG GTA CCG CCG AGG AAC CGC TAA CG and OED452:CAG GTA CCC GCG GAT GTG GAT GCA GC. Vector pW+SN attB was modified with a GAL4 sequence cloned as a KpnI/NotI fragment.*Khc-73* promoter was subsequently inserted into the unique KpnI site. Genomic *Khc-73* is from BACPAC clone CH321-36I16 (BACPAC Resources Center).

### Transgenic strains

Transgenic fly *CH321-36I16* was made by standard embryo injection of BACPAC clone CH321-36I16 (BACPAC Resources Center) with ΦC31 –mediated integration into attP site at position 86F of chromosome III.

### Electron microscopy

Wandering third instar larvae were dissected, prepared and embedded as described in [76]. Ultra-thin serial sections of 50 nm thickness were cut from muscle 6, 7 and 12 of hemisegment A3. Electron micrographs were taken at a magnification of 25,000x for measurements, 25,000x and 40,000x for figures. Serial Reconstruction and analysis was conducted on FIJI (Fiji is Just ImageJ) (NIH) [77] and Reconstruct v.1.1.0.0 Software [78].

### Immunostaining

Wandering third instar larvae were dissected as previously described [74]. Third Instar larvae were dissected in cold HL3 and fixed with 4% Paraformaldehyde for 10 min or 5min ice cold Methanol for GluRIIA staining. Larvae were washed with PBS (Phosphate buffered saline), permeabilized with PBT (PBS with 0.1% Triton X-100), blocked with 5% Normal Goat Serum (NGS) in PBT and placed in primary antibody overnight at 4°C. The larvae were then washed three times for 15min in PBT, placed in secondary antibody for 2 hrs, washed three times for 15min with PBT and mounted in Vectashield (Vector labs). Antibodies used are as follows: anti-GluRIII (1:500) (gift from A. DiAntonio, Washington Univ. St. Louis, MO), anti-Hrs (1:200), anti-SYT (1:1000) (gift from H. Bellen, Baylor College of Medicine, Houston, TX), anti-pMAD (PS1)(1:200) (gift from M.B. O’Connor, University of Minnesota, Minneapolis, MN). anti-Dlg (1:500), anti-nc82 (1:500), anti-GluRIIA(1:500), anti-CSP(1:500), anti-EPS15 (1:50), anti-LacZ (1:100) and anti-Wit (1:10) (Developmental Studies Hybridoma Bank(DSHB)), anti-HA (1:500) (HA.11 clone 16B12) (Covance Research Products), anti-GFP (1:500) (A6455) (Molecular Probes), anti-GFP (1:500) (Rat IgG2a, GF090R)(Nacalai Tesque Inc.), anti-HRP conjugated Alexa 647(1:250) (Jackson ImmunoResearch), anti-acetylated tubulin (1:500) (T7451, clone 6-11B-1 Sigma-Aldrich) and anti-pSmad3 (EP823Y)(Epitomics).

### Western blot analysis

Western blots were performed as previously described [41]. Muscle tissue (without the nervous system and motor axons or imaginal discs) or Brain tissue (VNC and axons) were isolated from wandering third instar larvae dissected in cold HL3. Western blot analysis was performed according to manufacturer’s protocols. Antibodies used: anti-Khc-73 (1:2000)[35], anti-Wit (1:10) (DSHB), anti-actin (Millipore, MAB1501). Gel images were scanned and band intensities were quantified using FIJI (Fiji is just ImageJ software) (NIH) [77].

### Confocal imaging and image analysis

Synapses were imaged using a ConfoCor LSM710 and Zeiss LSM 780 on an Axiovert 200M inverted microscope (Carl Zeiss, Inc.) with 63x/1.4 oil objective. Image analysis was performed with ImageJ 1.46j (NIH) [79], Imaris (Bitplane Scientific Software), Image Analyst MKII (Image Analyst Software, Novato, CA) and Metamorph (Molecular Devices).

### Live imaging

Wandering third instar larvae were dissected in room temperature HL3 to remove the guts and fat bodies. The larval filet was then inverted and stretched onto a coverslip using magnetic dissection pins inside a chamber consisting of a coverslip surrounded by magnet strips. Larval prep was maintained at room temperature in an HL3 bath during imaging. NMJs at hemisegment A3 and A4, muscles 6/7 and 4 were imaged. Axons were imaged at hemisegment A3 to A4. Larvae were imaged for a maximum of 30 minutes after dissection. Axons and NMJs were imaged with 63x 1.4NA oil objective on Axiovert 200 inverted microscope with Zeiss LSM780 confocal (Carl Zeiss, Inc.).

### Nerve crush assay

The nerve crush assay was performed as previously described [65]. Briefly, third instar larvae were anaesthetized with carbon dioxide. The segmental nerves at the midbody were then pinched with Dumostar number 5 forceps for five seconds. The larvae were then recovered on standard media for 25 hours at 25°C after which time they were dissected and stained for LacZ.

### Electrophysiology

Wandering third instar larvae were dissected in cold HL3 solution following standard protocol [80]. The spontaneous (mEJC) and evoked (EJC) membrane currents were recorded from muscle 6 in abdominal segment A3 with standard two-electrode voltage-clamp technique [41]. All the recordings were performed at room temperature in HL3 solution containing 0.5mM Ca2+ unless otherwise indicated. The current recordings were collected with AxoClamp2B amplifier (Molecular Devices Inc.) using Clampex 9.2 software (Molecular Devices Inc.). The nerve stimulation was delivered through a suction electrode which held the cut nerve terminal cord. In all voltage clamp recordings, muscles were held at -80 mV. The holding current was less than 5 nA for 90% of the recordings and we rejected any recording that required more than 10 nA current to maintain the holding potential.

The amplitudes of mEJC and EJC were measured using Mini Analysis 6.0.3 software (Synaptosoft) and verified by eye. QC was calculated by dividing the mean EJC amplitude by mean mEJC amplitude. The recording traces were generated with Origin 7.5 software (Origin Lab).Spontaneous and evoked potentials were measured as previously described [49]. Standard two-electrode voltage-clamp technique was used as described in [44].

## Supporting information

S1 Fig *Khc-73* mutants, and synaptic phenotypes. (A) Khc-73 protein domain structure in *D*. *melanogaster* and *H*. *sapiens*. K (Kinesin motor), FHA (Forkhead Associated), CC (coiled coil) and CAP-GLY (Cytoskeletal Associated Proteins–Glycine Rich) domains. (B) *Khc-73* gene structure and location of DP00530 insert. Untranslated regions (UTR) are in solid black. Exons in grey. Deletions *Khc-73*^*193*^ and *Khc-73*^*149*^ are indicated by solid lines. (C) Western blot of Control (*Khc-73*^*100*^), *Khc-73* heterozygotes (*Khc-73*^*149*^/+), *Khc-73* mutants (*Khc-73*^*149*^) (left) and Control (*Khc-73*^*100*^), *Khc-73*^*193*^ (right) with anti-Khc-73 (top) and actin loading control (bottom).
(D) Localization of HA-Khc-73 in axons (*BG380-Gal4*/+; *UAS-HA-Khc-73*). Scale bar is 5μm.
(E) Localization of HA-Khc-73 in muscle 4 NMJ terminal boutons (*BG380-Gal4*/+; *UAS-HA-Khc-73*). Scale bar is 5μm. (F) Expression of *Khc-73*-GAL4 visualized with mCD8:GFP in ventral nerve cord (*Khc-73-Gal4*/*UAS-mCD8*:*GFP*). Scale bar is 100μm. (G) Expression of *Khc-73-GAL4* visualized with mCD8:GFP in muscle 4 NMJ (*Khc-73-Gal4*/*UAS-mCD8*:*GFP*). Scale bar is 5μm. (H) Expression pattern of *Khc-73-GAL4* visualized with mCD8:GFP in the adult CNS and VNC. (I) Quantification of bouton number in Control (*Khc-73*^*100*^) and *Khc-73* (*Khc-73*^*149*^) mutants at muscle 6/7. N = 17, 18 NMJs. Error Bars are SEM. (J) Muscle surface area of muscle 6/7 in Control (*Khc-73*^*100*^) and *Khc-73* (*Khc-73*^*149*^). Muscle 6/7 normalized to control. n = 17, 18. Error Bars are SEM. Student’s t-test. ns-no statistical significance. (K) High Frequency Stimulation trace for Control (*Khc-73*^*100*^) and *Khc-73* (*Khc-73*^*193*^). N = 11, 13 NMJs. (L) Quantification of EJP amplitudes for genotypes in (K). Recording duration was 600 seconds at 10Hz. EJPs were binned per 30 seconds and amplitudes were averaged per bin. Relative EJP amplitudes were normalized to the average amplitude for the first 15seconds of recording for each genotype. Error bars are SEM. N = 9,8.
(TIF)

S2 Fig *Khc-73* synaptic phenotypes. (A) Muscle 4 terminal boutons stained with anti-synaptotagmin (SYT). Scale is 5μm. (B) Quantification of SYT mean fluorescence intensity normalized to HRP signal and expressed as a percentage of control for muscle 4 boutons in control (*Khc-73*^*193*^/+) and *Khc-73* (*Khc-73*^*193*^*/Khc-73*^*193*^). N = 10, 8 NMJs. (C) Quantification of Cysteine String Protein (CSP) mean fluorescence intensity, normalized to HRP intensity and expressed as a percentage of control, of muscle 4 NMJs. Control (*Khc-73*^*100*^) and *Khc-73* (*Khc-73*^*193*^), N = 9, 10. (D) Quantification of EPS-15 staining in control (*w*^*1118*^) and *Khc-73* (*Khc-73*^*149*^) larval NMJs. N = 6, 6. (E) Muscle 4 terminal boutons stained with Dlg in control (*Khc-73*^*100*^) (top) and *Khc-73* (*Khc-73*^*193*^) (bottom) third instar larvae. Scale bar is 5μm. (F) Quantification of Dlg fluorescence intensity normalized to HRP intensity and expressed as a percentage of control.
(G) Quantification of GluRIIA fluorescence intensity normalized to HRP intensity and expressed as a percentage of control for Control (*Khc-73*^*100*^) and *Khc-73* (*Khc-73*^*149*^) mutants. Error Bars are SEM. Student’s t-test. ***P<0.001, ns-no statistical significance.
(TIF)

S3 Fig *Khc-73* genetic interaction with BMP pathway. (A) Brp puncta in terminal boutons of muscle 4 NMJs in Control (*Khc-73*^*100*^/+), *Medea* (*Medea*^*C246*^/*Medea*^*G112*^) and *Khc-73*; *Medea* (*Khc-73*^*149*^; *Medea*^*C246*^ */ Medea*^*G112*^) larvae. (B) Quantification of BRP puncta number for genotypes in (A). N = 19,20,20 NMJs. (C) Quantification of synaptic area identified with HRP staining for genotypes in (A). N = 19,20,20 NMJs. (D) Quantification of bouton number per NMJ for genotypes in (A). N = 19,20,20 NMJs. (E) Brp puncta in axons of Control (*Khc-73*^*100*^/+), *Medea* (*Medea*^*C246*^/*Medea*^*G112*^) and *Khc-73*; *Medea* (*Khc-73*^*149*^; *Medea*^*C246*^ */ Medea*^*G112*^) larvae. Green channel–Brp, Red channel–HRP. (F) Quantification of Brp puncta axon density for genotypes in (E) normalized to control. N = 10,10 and 10 Larvae. (G) Acetylated tubulin staining in muscle 4 NMJs of Control (*Khc-73*^*100*^/+) and *Khc-73* (*Khc-73*^*149*^), Dashed line–Quantified region. Acetylated-tubulin (green) and HRP (red). Scale bar is 10μm. (H) Quantification of Acetylated-tubulin intensity in axon region indicated by dashed line in (G) N = 9,8. Error Bars are SEM. Student’s t-test. *P<0.05, **P<0.01, ***P<0.001. ns-no statistical significance. Scale bar is 5μm.
(TIF)

S4 Fig Quantification of pMAD levels in larvae overexpressing Khc-73. (A) Quantification of pMad intensity at muscle 4 NMJs for control (*Khc-73*^*100*^), *Khc-73*^*149*^ and *Khc-73*^*193*^. N = 10, 12 and 15 NMJs. (B) Quantification of pMad intensity in VNC neurons for control (*w*^*1118*^) and *Khc-73* mutants (*Khc-73*^*149*^). N = 6(217), 6(234), VNCs(nuclei). (C) Quantification of pMAD intensity at the NMJ in larvae overexpressing Khc-73 in motoneurons. Control (*OK371-Gal4*/+) and Khc-73 OE (*OK371-Gal4*/*UAS-Khc-73*). N = 20, 19. (D) Quantification of pMAD intensity in the ventral nerve cord in larvae overexpressing Khc-73 in motoneurons. Control (*OK371-Gal4*/+) and Khc-73 OE (*OK371-Gal4*/UAS-*Khc-73*) N = 4(100), 3(143), larvae (nuclei).Error Bars are SEM. Student’s t-test. ns-no statistical significance.
(TIF)

S5 Fig BMP receptors accumulate at NMJs in *Khc-73* mutant larvae. (A) Image of muscle 4 NMJs in live, unfixed larvae for Control (*BG380-Gal4*/+; *OK371-Gal4*/+; *UAS-TKV-YFP*/+) and *Khc-73* (*BG380-Gal4*/+; *Khc-73*^*149*^,*OK371-Gal4*/ *Khc-73*^*149*^; *UAS-TKV-YFP*/+). Scale bar is 10μm. (B) Quantification of mean fluorescence intensity as percentage of control for genotypes in (A). N = 14, 12 NMJs. (C) Live image of muscle 6/7 NMJs in live, unfixed larvae for Control (*BG380-Gal4*/+; *OK371-Gal4*/+; *UAS-TKV-YFP*/+) and *Khc-73* (*BG380-Gal4*/+; *Khc-73*^*149*^,*OK371-Gal4*/ *Khc-73*^*149*^; *UAS-TKV-YFP*/+). Scale bar is 10μm. (D) Quantification of mean fluorescence intensity as percentage of control for genotypes in (C). N = 15, 15 NMJs.
(E) Quantitative PCR analysis of *UAS-TKV-YFP* mRNA expression levels in *Khc-73* mutants. N = 3 technical replicates. (F) Kymograph of TKV-YFP live imaging of axons in third instar larvae in control (*BG380-Gal4*/+; *OK371-Gal4*/+; *UAS-TKV-YFP*/+) and *Khc-73* mutants (*BG380-Gal4*/+; *Khc-73*^*149*^ /*Khc-73*^*149*^, *OK371-Gal4*; *UAS-TKV-YFP*/+) larvae. Scale bar is 10μm. Error Bars are SEM. Student’s t-test. ***P<0.001. ns-not statistically significant.
(TIF)

S6 Fig MVB marker Hrs is increased in *Khc-73* mutant boutons overexpressing Wit. (A) Muscle 4 terminal bouton in Wit OE Control (*OK371-Gal4*, *Khc-73*^*149*^*/UAS-Wit*) and Wit OE, *Khc-73* larvae (*OK371-Gal4*, *Khc-73*^*149*^*/UAS-Wit*, *Khc-73*^*149*^) stained for Hrs (white) and Hrp (red). Scale bar is 5μm. (B) Quantification of Hrs staining in (A). N = 20, 20 NMJs. Error Bars are SEM. *P<0.05. Student’s t-test.
(TIF)

S7 Fig Wit colocalizes with TKV and Rab7:GFP at the NMJ. (A) Muscle 4 terminal boutons in motoneurons overexpressing Wit and Rab7:GFP (*nSyb-Gal4*, *UAS-TKV-YFP/UAS-Rab7*:*GFP*) stained with Wit (Green) and GFP (red) antibodies. Scale bar is 5μm. (B) Muscle 4 terminal boutons in motoneurons overexpressing Wit and TKV-YFP (*UAS-Wit/+; nSyb-Gal4*, *UAS-TKV-YFP/+*) stained with Wit (Green) and GFP (red) antibodies. Scale bar is 5μm.
(TIF)

S8 Fig Axonal transport in *Khc-73* mutants is normal. (A) RAB7:GFP expressed in live third instar larval axons of Control (*BG380-Gal4*/+; *UAS-RAB7*:*GFP*/+) and *Khc-73* mutants (*BG380-Gal4*/+; *Khc-73*^*149*^; *UAS-RAB7*:*GFP*/+). Scale bar is 5 μm. (B) Kymographs from live imaging of RAB7:GFP in axons of Control (*BG380-Gal4*/+; *UAS-RAB7*:*GFP*/+) and *Khc-73* mutants (*BG380-Gal4*/+; *Khc-73*^*149*^; *UAS-RAB7*:*GFP*/+). (C) Histogram of Retrograde velocities of RAB7:GFP puncta in Control (*BG380-Gal4*/+; *UAS-RAB7*:*GFP*/+) and *Khc-73* mutants (*BG380-Gal4*/+; *Khc-73*^*149*^; *UAS-RAB7*:*GFP*/+). n = 5, 8 axons and n = 64, 49 puncta respectively.
(D) Histogram of anterograde velocities of RAB7:GFP puncta in Control and *Khc-73* mutants. n = 5, 8 axons and n = 49, 27 puncta respectively. (E) LacZ staining in control (*Khc73*^*100*^; *puc*^*E69*^ /+) and *Khc-73* mutant (*Khc-73*^*149*^; *puc*^*E69*^ /+) larval motor neuron nuclei in the ventral nerve cord. Scale bar is 10μm. (F) Quantification of mean LacZ fluorescence staining in nuclei for genotypes in (E). N = 279, (6) for control (*Khc73*^*100*^; *puc*^*E69*^ /+) and 535, (11) for *Khc-73* mutant (*Khc-73*^*149*^; *puc*^*E69*^ /+), Nuclei, (Ventral Nerve Cords), respectively. (G) LacZ staining in control (*Khc73*^*100*^*; puc*^*E69*^ /+) and *Khc-73* mutant (*Khc-73*^*149*^; *puc*^*E69*^ /+) larval motor neuron nuclei in the ventral nerve cord after nerve crush assay. Scale bar is 10μm. (H) Quantification of Anterior and Posterior mean fluorescence of nuclei in (G) expressed as Posterior/Anterior ratio. N = 95, 113, (4) for *Khc-73*^*100*^*; puc*^*E69*^ /+. 219, 300, (10) for *Khc-73*^*149*^*; puc*^*E69*^ /+. Anterior nuclei, posterior nuclei, (Ventral Nerve Cords), respectively. Error Bars are SEM. Student’s t-test. ns-no statistical significance.
(TIF)

S1 Movie Rab7:GFP puncta at muscle 4 NMJ control. Live imaging of Rab7:GFP in third instar larva, muscle 4 NMJ in control (*OK371-GAL4*/+; *UAS-RAB7*:*GFP/+*). Retrograde direction (toward motoneuron cell body) is to the left. Scale bar is 5 μm.
(GIF)

S2 Movie Rab7:GFP puncta at muscle 4 NMJ in *Khc-73* mutant. Live imaging of Rab7:GFP in third instar larva, muscle 4 NMJ in *Khc-73* mutant (*OK371-GAL4*, *Khc-73*^*149*^/+, *Khc-73*^*149*^; *UAS-RAB7*:*GFP*/+). Retrograde direction (toward motoneuron cell body) is to the left. Scale bar is 5 μm.
(GIF)

S3 Movie Rab7:GFP puncta in motoneuron axons of control. Live imaging of Rab7:GFP in third instar larva axon in control (*OK371-GAL4*/+; *UAS-RAB7*:*GFP/+*). Retrograde direction (toward motoneuron cell body) is to the left. Scale bar is 10 μm.
(GIF)

S4 Movie Rab7:GFP puncta in motoneuron axons of *Khc-73* mutant. Live imaging of Rab7:GFP in third instar larva axon in *Khc-73* mutant (*OK371-GAL4*, *Khc-73*^*149*^/+, *Khc-73*^*149*^; *UAS-RAB7*:*GFP*/+). Retrograde direction (toward motoneuron cell body) is to the left. Scale bar is 10 μm.
(GIF)

S5 Movie TKV:YFP puncta in muscle 4 NMJ of control. Live imaging of TKV:YFP in third instar larva, muscle 4 NMJ in control (*BG380-Gal4*/+; *OK371-Gal4*/+; *UAS-TKV-YFP*/+). Retrograde direction (toward motoneuron cell body) is to the left. Scale bar is 5 μm.
(GIF)

S6 Movie TKV:YFP puncta in muscle 4 NMJ of *Khc-73* mutant. Live imaging of TKV:YFP in third instar larva, muscle 4 NMJ in *Khc-73* mutant (*BG380-Gal4*/+; *Khc-73*^*149*^,*OK371-Gal4*/ *Khc-73*^*149*^; *UAS-TKV-YFP*/+). Retrograde direction (toward motoneuron cell body) is to the left. Scale bar is 5 μm.
(GIF)

S7 Movie Mito:GFP in axons of control. Live imaging of axons in third instar larva of control (*OK371-GAL4*/*UAS-mito*:*GFP*). Retrograde direction is to the left. Scale bar is 10μm.
(GIF)

S8 Movie Mito:GFP in axons of *Khc-73* mutants. Live imaging of axons in third instar larva of *Khc-73* mutant (*OK371-GAL4*, *Khc-73*^*149*^/*UAS-mito*:*GFP*, *Khc-73*^*149*^). Retrograde direction is to the left. Scale bar is 10μm.
(GIF)
