# Supplementary figures and images for: Kinesin Khc-73/KIF13B modulates retrograde BMP signaling by influencing endosomal dynamics at the Drosophila neuromuscular junction
Source: PLoS Genet. 2018 Jan 26;14(1):e1007184. doi: 10.1371/journal.pgen.1007184 (PMC5802963; doi:10.1371/journal.pgen.1007184)

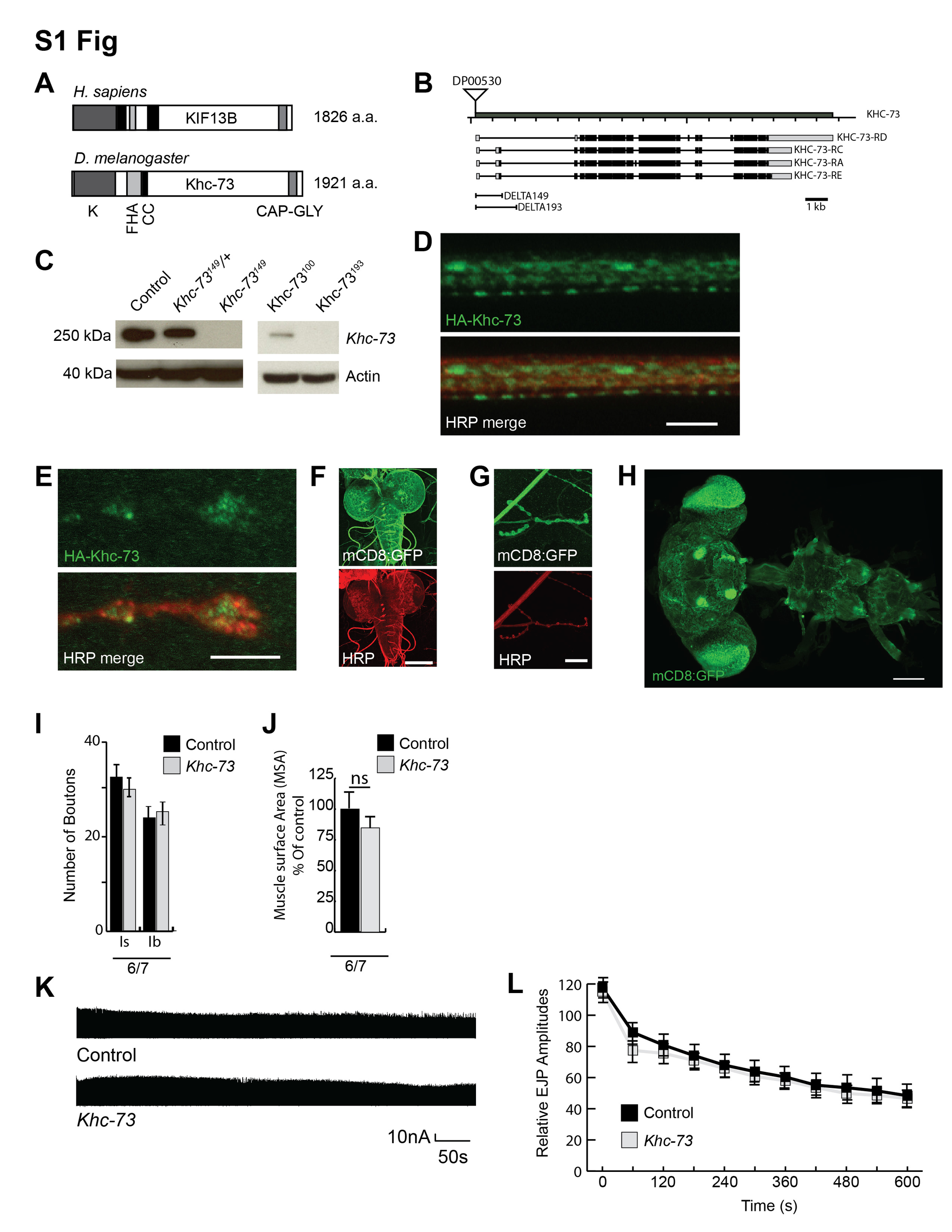

Supplement: S1 Fig — (A) Khc-73 protein domain structure in D. melanogaster and H. sapiens. K (Kinesin motor), FHA (Forkhead Associated), CC (coiled coil) and CAP-GLY (Cytoskeletal Associated Proteins–Glycine Rich) domains. (B) Khc-73 gene structure and location of DP00530 insert. Untranslated regions (UTR) are in solid black. Exons in grey. Deletions Khc-73193 and Khc-73149 are indicated by solid lines. (C) Western blot of Control (Khc-73100), Khc-73 heterozygotes (Khc-73149/+), Khc-73 mutants (Khc-73149) (left) and Control (Khc-73100), Khc-73193 (right) with anti-Khc-73 (top) and actin loading control (bottom). (D) Localization of HA-Khc-73 in axons (BG380-Gal4/+; UAS-HA-Khc-73). Scale bar is 5μm. (E) Localization of HA-Khc-73 in muscle 4 NMJ terminal boutons (BG380-Gal4/+; UAS-HA-Khc-73). Scale bar is 5μm. (F) Expression of Khc-73-GAL4 visualized with mCD8:GFP in ventral nerve cord (Khc-73-Gal4/UAS-mCD8:GFP). Scale bar is 100μm. (G) Expression of Khc-73-GAL4 visualized with mCD8:GFP in muscle 4 NMJ (Khc-73-Gal4/UAS-mCD8:GFP). Scale bar is 5μm. (H) Expression pattern of Khc-73-GAL4 visualized with mCD8:GFP in the adult CNS and VNC. (I) Quantification of bouton number in Control (Khc-73100) and Khc-73 (Khc-73149) mutants at muscle 6/7. N = 17, 18 NMJs. Error Bars are SEM. (J) Muscle surface area of muscle 6/7 in Control (Khc-73100) and Khc-73 (Khc-73149). Muscle 6/7 normalized to control. n = 17, 18. Error Bars are SEM. Student’s t-test. ns-no statistical significance. (K) High Frequency Stimulation trace for Control (Khc-73100) and Khc-73 (Khc-73193). N = 11, 13 NMJs. (L) Quantification of EJP amplitudes for genotypes in (K). Recording duration was 600 seconds at 10Hz. EJPs were binned per 30 seconds and amplitudes were averaged per bin. Relative EJP amplitudes were normalized to the average amplitude for the first 15seconds of recording for each genotype. Error bars are SEM. N = 9,8. (TIF) [file pgen.1007184.s001.tif]

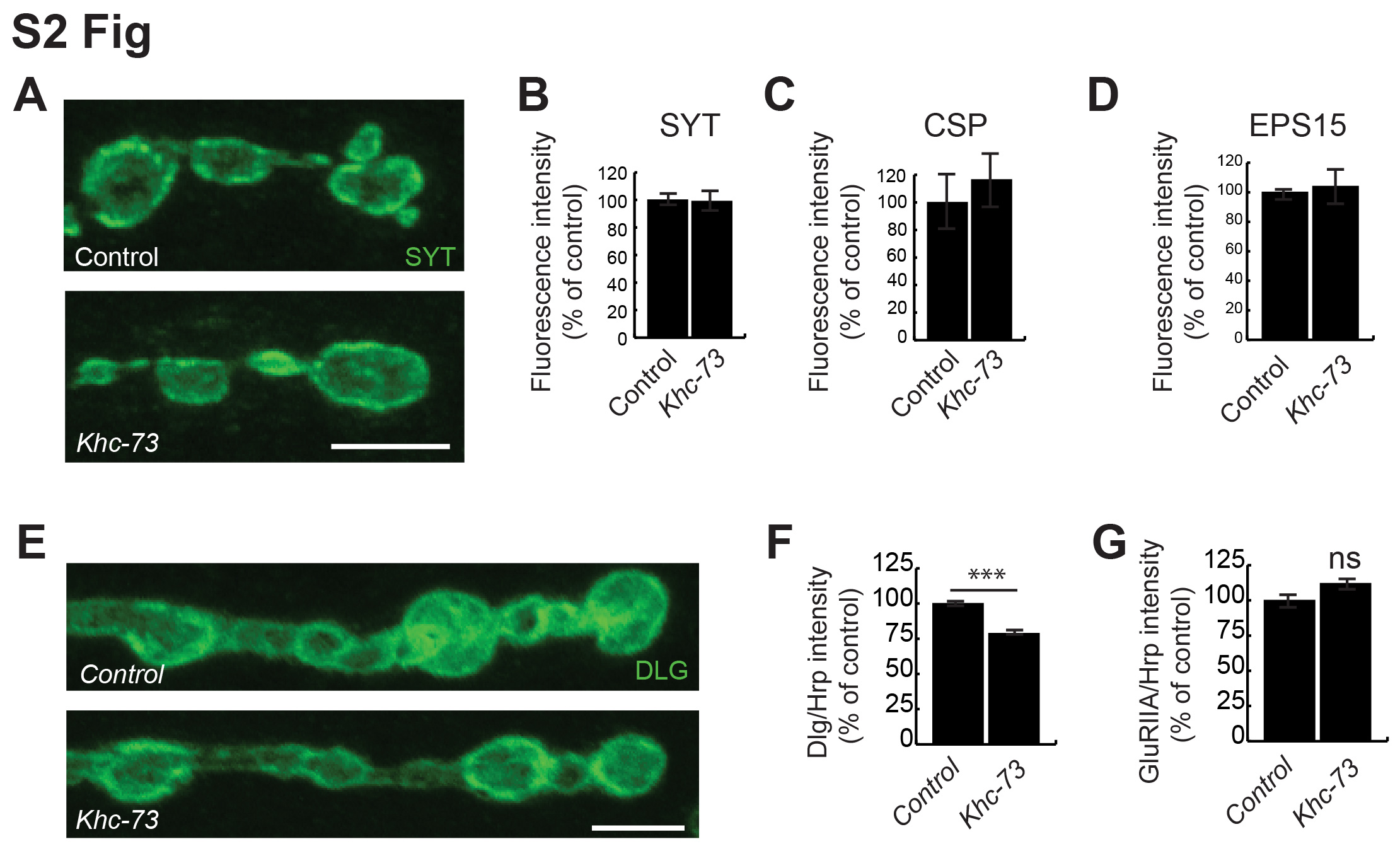

Supplement: S2 Fig — (A) Muscle 4 terminal boutons stained with anti-synaptotagmin (SYT). Scale is 5μm. (B) Quantification of SYT mean fluorescence intensity normalized to HRP signal and expressed as a percentage of control for muscle 4 boutons in control (Khc-73193/+) and Khc-73 (Khc-73193/Khc-73193). N = 10, 8 NMJs. (C) Quantification of Cysteine String Protein (CSP) mean fluorescence intensity, normalized to HRP intensity and expressed as a percentage of control, of muscle 4 NMJs. Control (Khc-73100) and Khc-73 (Khc-73193), N = 9, 10. (D) Quantification of EPS-15 staining in control (w1118) and Khc-73 (Khc-73149) larval NMJs. N = 6, 6. (E) Muscle 4 terminal boutons stained with Dlg in control (Khc-73100) (top) and Khc-73 (Khc-73193) (bottom) third instar larvae. Scale bar is 5μm. (F) Quantification of Dlg fluorescence intensity normalized to HRP intensity and expressed as a percentage of control. (G) Quantification of GluRIIA fluorescence intensity normalized to HRP intensity and expressed as a percentage of control for Control (Khc-73100) and Khc-73 (Khc-73149) mutants. Error Bars are SEM. Student’s t-test. ***P<0.001, ns-no statistical significance. (TIF) [file pgen.1007184.s002.tif]

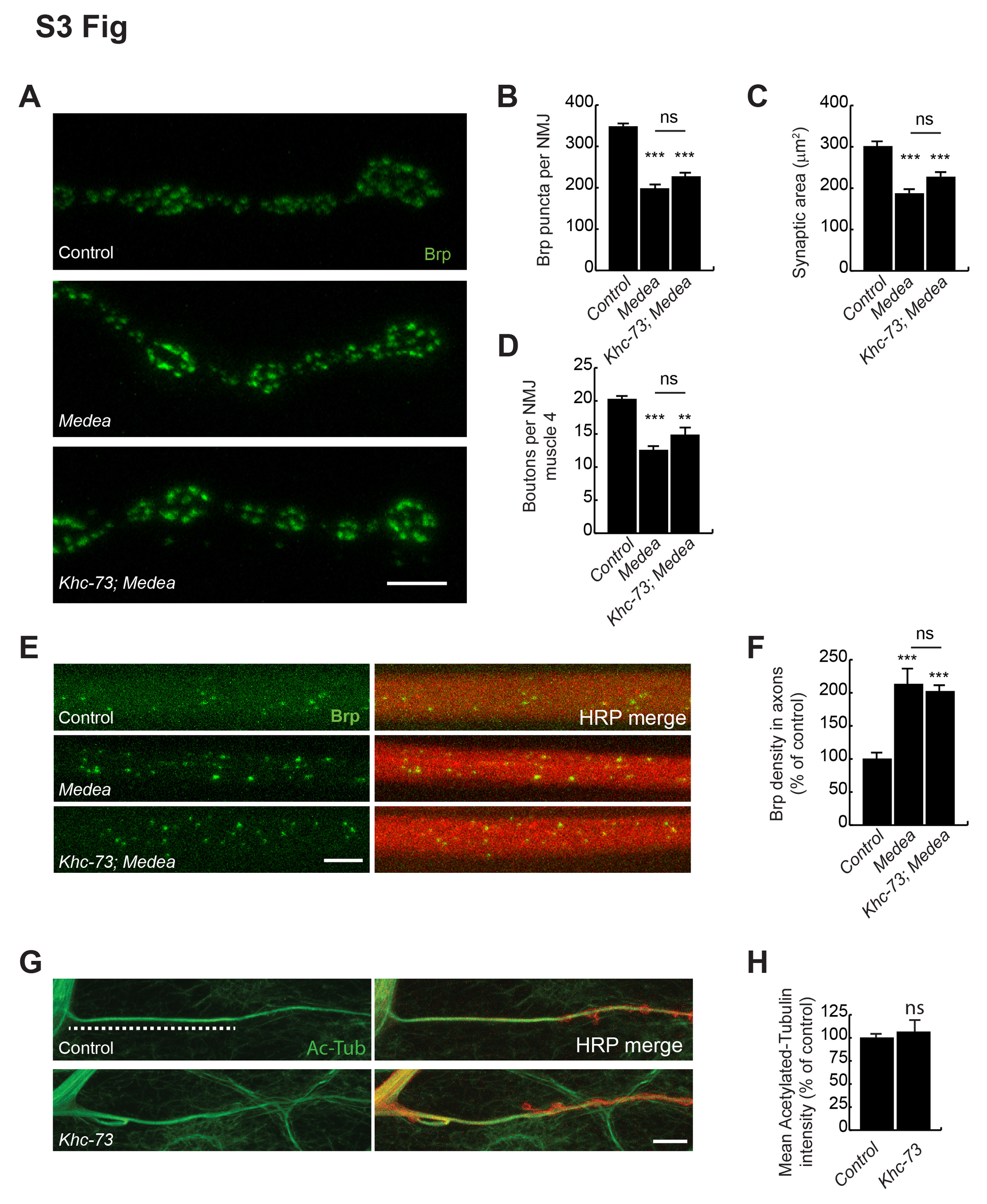

Supplement: S3 Fig — (A) Brp puncta in terminal boutons of muscle 4 NMJs in Control (Khc-73100/+), Medea (MedeaC246/MedeaG112) and Khc-73; Medea (Khc-73149; MedeaC246 / MedeaG112) larvae. (B) Quantification of BRP puncta number for genotypes in (A). N = 19,20,20 NMJs. (C) Quantification of synaptic area identified with HRP staining for genotypes in (A). N = 19,20,20 NMJs. (D) Quantification of bouton number per NMJ for genotypes in (A). N = 19,20,20 NMJs. (E) Brp puncta in axons of Control (Khc-73100/+), Medea (MedeaC246/MedeaG112) and Khc-73; Medea (Khc-73149; MedeaC246 / MedeaG112) larvae. Green channel–Brp, Red channel–HRP. (F) Quantification of Brp puncta axon density for genotypes in (E) normalized to control. N = 10,10 and 10 Larvae. (G) Acetylated tubulin staining in muscle 4 NMJs of Control (Khc-73100/+) and Khc-73 (Khc-73149), Dashed line–Quantified region. Acetylated-tubulin (green) and HRP (red). Scale bar is 10μm. (H) Quantification of Acetylated-tubulin intensity in axon region indicated by dashed line in (G) N = 9,8. Error Bars are SEM. Student’s t-test. *P<0.05, **P<0.01, ***P<0.001. ns-no statistical significance. Scale bar is 5μm. (TIF) [file pgen.1007184.s003.tif]

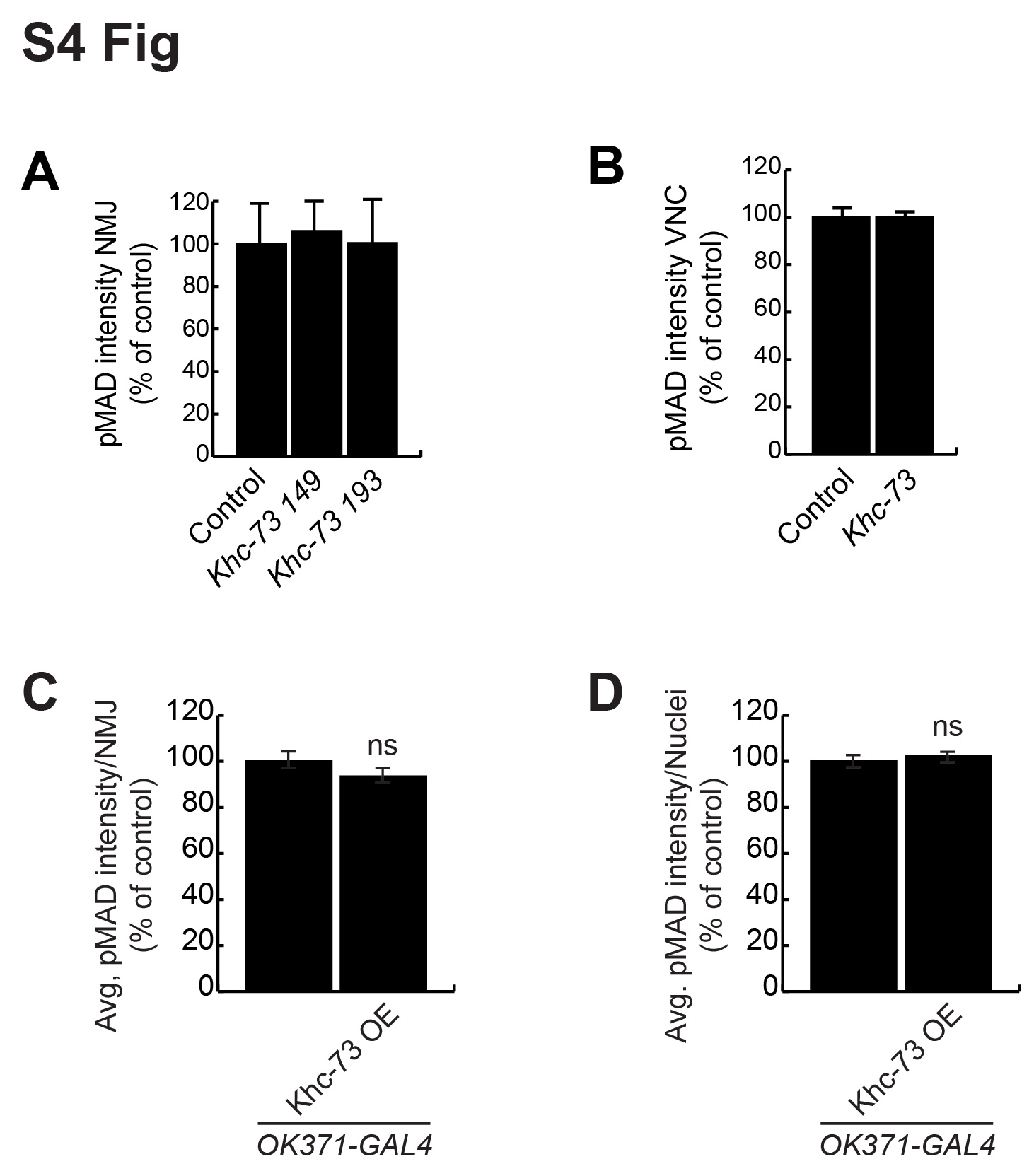

Supplement: S4 Fig — (A) Quantification of pMad intensity at muscle 4 NMJs for control (Khc-73100), Khc-73149 and Khc-73193. N = 10, 12 and 15 NMJs. (B) Quantification of pMad intensity in VNC neurons for control (w1118) and Khc-73 mutants (Khc-73149). N = 6(217), 6(234), VNCs(nuclei). (C) Quantification of pMAD intensity at the NMJ in larvae overexpressing Khc-73 in motoneurons. Control (OK371-Gal4/+) and Khc-73 OE (OK371-Gal4/UAS-Khc-73). N = 20, 19. (D) Quantification of pMAD intensity in the ventral nerve cord in larvae overexpressing Khc-73 in motoneurons. Control (OK371-Gal4/+) and Khc-73 OE (OK371-Gal4/UAS-Khc-73) N = 4(100), 3(143), larvae (nuclei).Error Bars are SEM. Student’s t-test. ns-no statistical significance. (TIF) [file pgen.1007184.s004.tif]

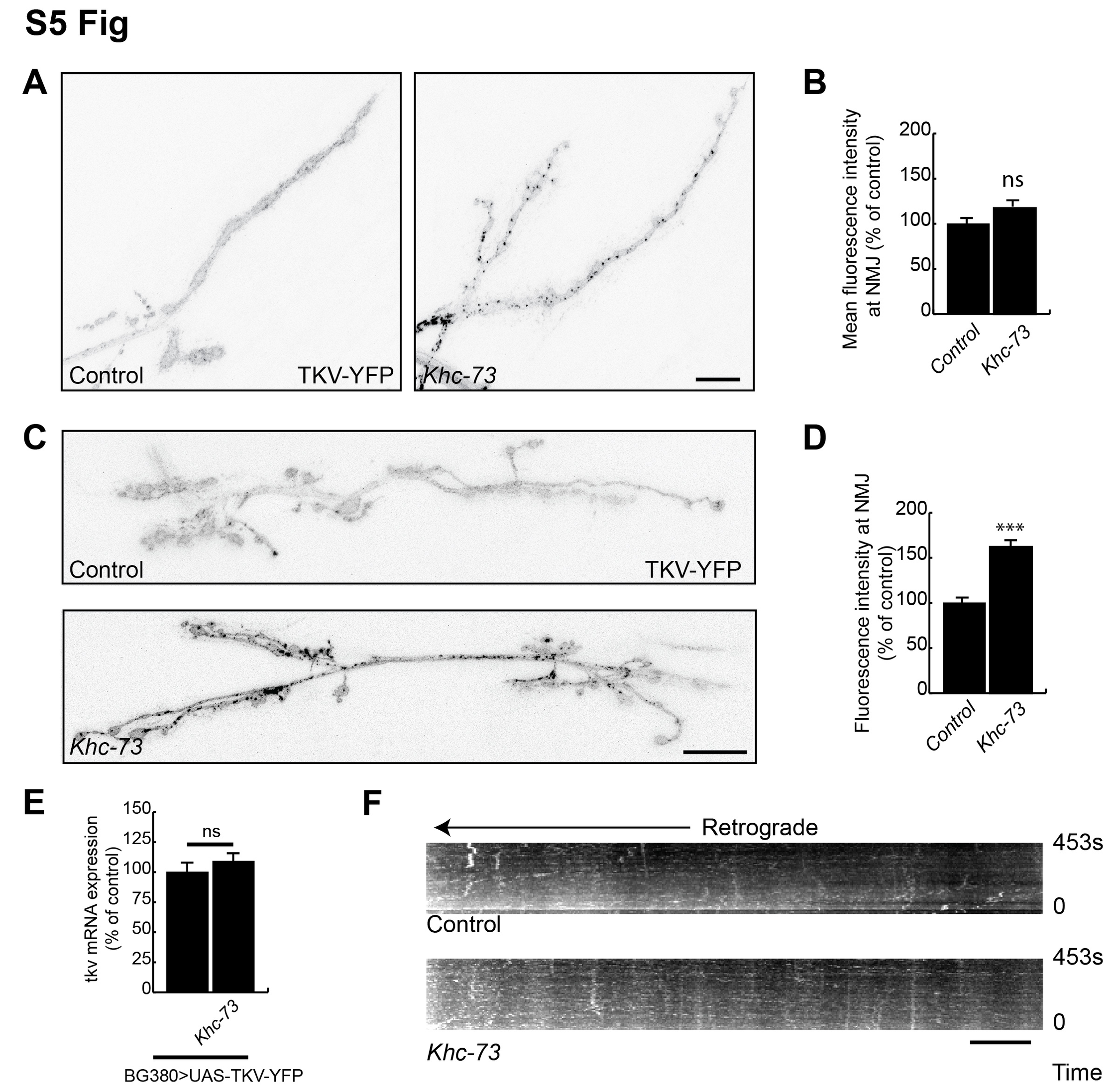

Supplement: S5 Fig — (A) Image of muscle 4 NMJs in live, unfixed larvae for Control (BG380-Gal4/+; OK371-Gal4/+; UAS-TKV-YFP/+) and Khc-73 (BG380-Gal4/+; Khc-73149,OK371-Gal4/ Khc-73149; UAS-TKV-YFP/+). Scale bar is 10μm. (B) Quantification of mean fluorescence intensity as percentage of control for genotypes in (A). N = 14, 12 NMJs. (C) Live image of muscle 6/7 NMJs in live, unfixed larvae for Control (BG380-Gal4/+; OK371-Gal4/+; UAS-TKV-YFP/+) and Khc-73 (BG380-Gal4/+; Khc-73149,OK371-Gal4/ Khc-73149; UAS-TKV-YFP/+). Scale bar is 10μm. (D) Quantification of mean fluorescence intensity as percentage of control for genotypes in (C). N = 15, 15 NMJs. (E) Quantitative PCR analysis of UAS-TKV-YFP mRNA expression levels in Khc-73 mutants. N = 3 technical replicates. (F) Kymograph of TKV-YFP live imaging of axons in third instar larvae in control (BG380-Gal4/+; OK371-Gal4/+; UAS-TKV-YFP/+) and Khc-73 mutants (BG380-Gal4/+; Khc-73149 /Khc-73149, OK371-Gal4; UAS-TKV-YFP/+) larvae. Scale bar is 10μm. Error Bars are SEM. Student’s t-test. ***P<0.001. ns-not statistically significant. (TIF) [file pgen.1007184.s005.tif]

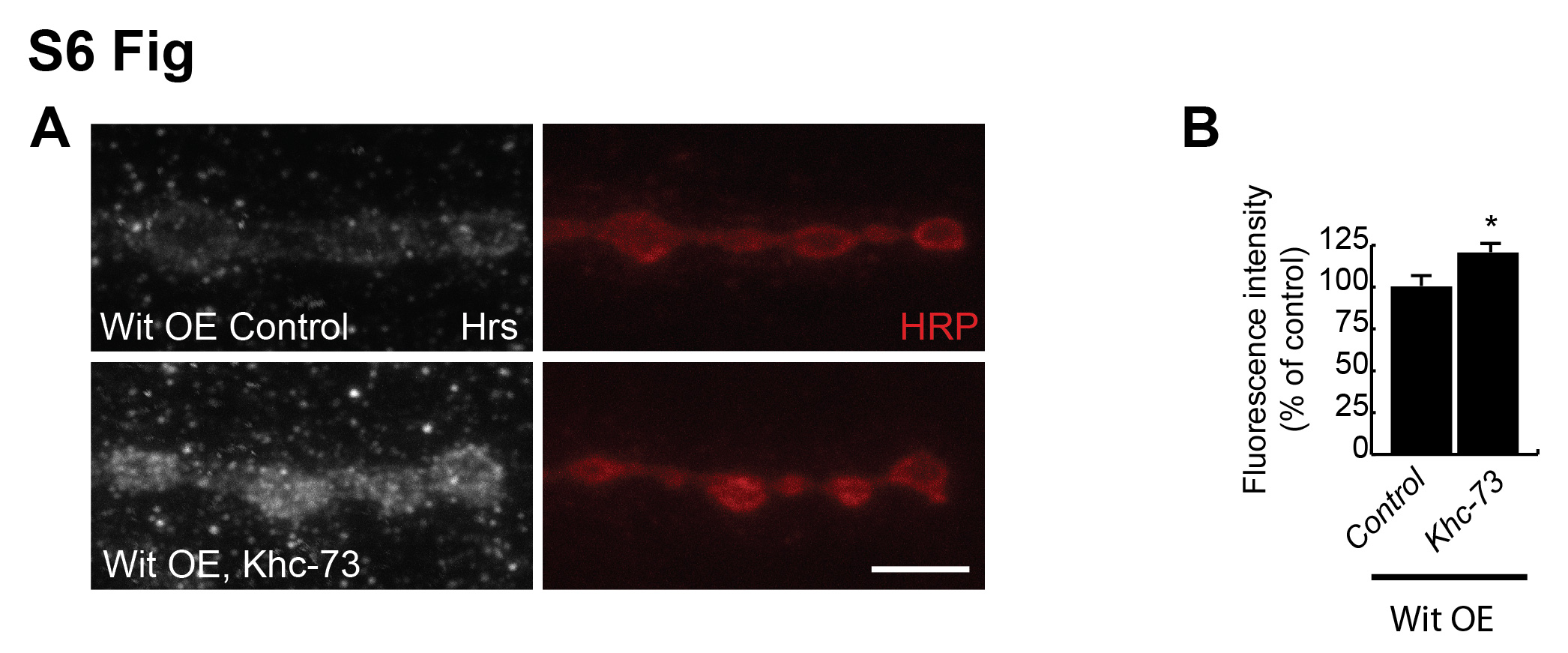

Supplement: S6 Fig — (A) Muscle 4 terminal bouton in Wit OE Control (OK371-Gal4, Khc-73149/UAS-Wit) and Wit OE, Khc-73 larvae (OK371-Gal4, Khc-73149/UAS-Wit, Khc-73149) stained for Hrs (white) and Hrp (red). Scale bar is 5μm. (B) Quantification of Hrs staining in (A). N = 20, 20 NMJs. Error Bars are SEM. *P<0.05. Student’s t-test. (TIF) [file pgen.1007184.s006.tif]

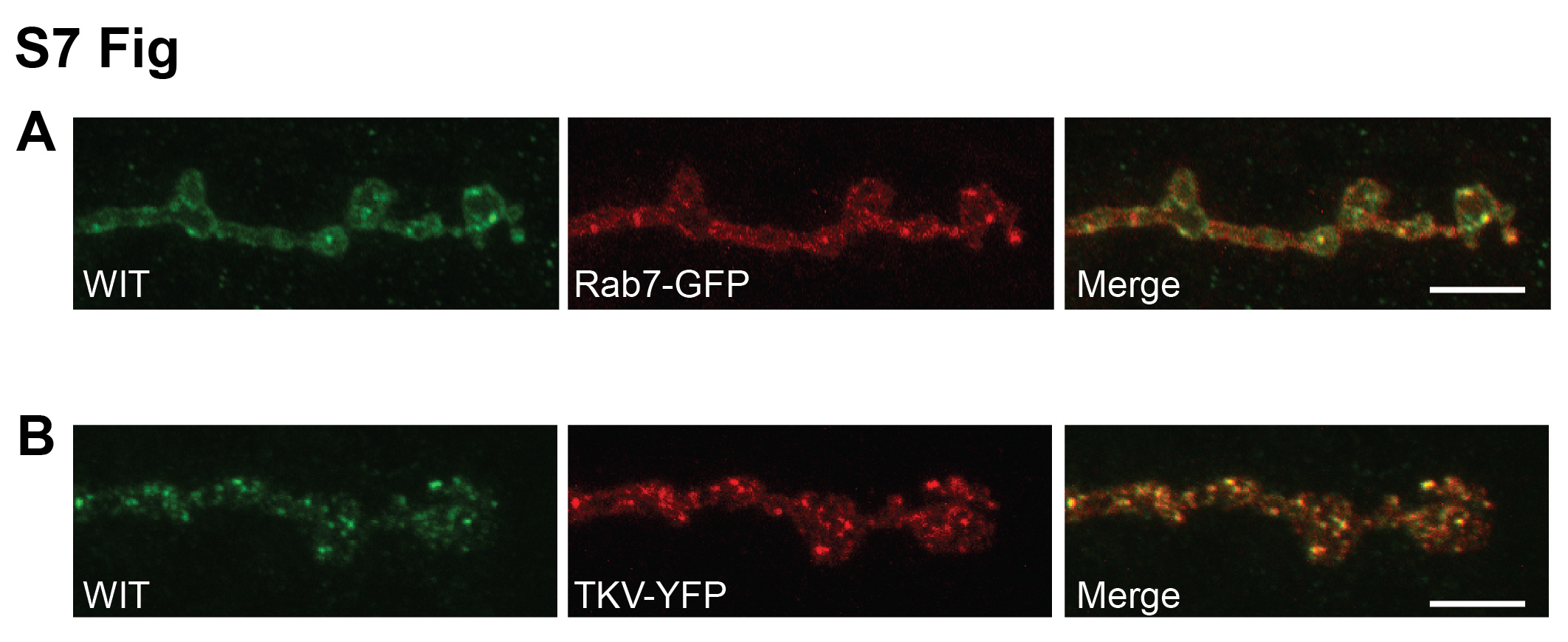

Supplement: S7 Fig — (A) Muscle 4 terminal boutons in motoneurons overexpressing Wit and Rab7:GFP (nSyb-Gal4, UAS-TKV-YFP/UAS-Rab7:GFP) stained with Wit (Green) and GFP (red) antibodies. Scale bar is 5μm. (B) Muscle 4 terminal boutons in motoneurons overexpressing Wit and TKV-YFP (UAS-Wit/+; nSyb-Gal4, UAS-TKV-YFP/+) stained with Wit (Green) and GFP (red) antibodies. Scale bar is 5μm. (TIF) [file pgen.1007184.s007.tif]

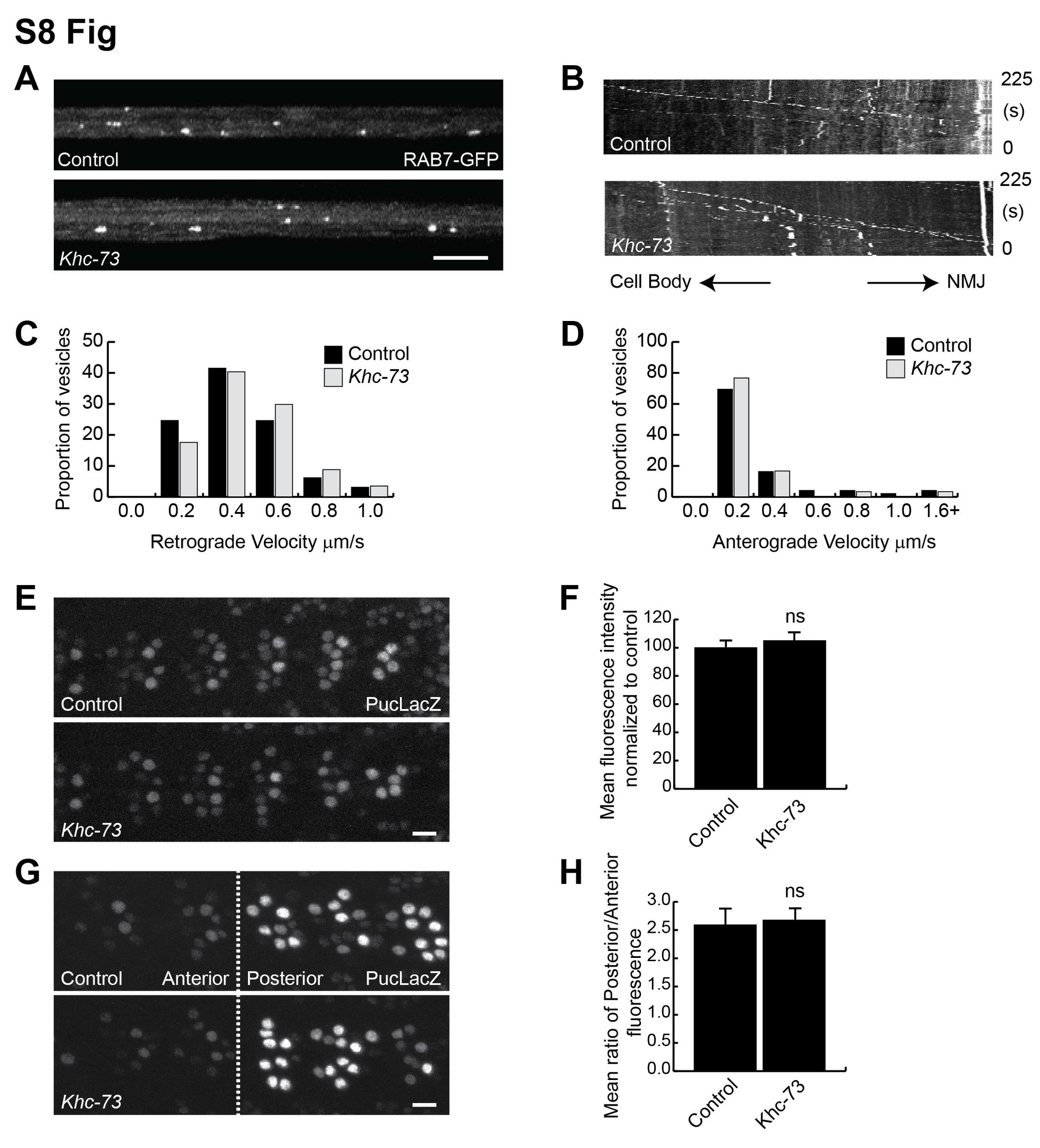

Supplement: S8 Fig — (A) RAB7:GFP expressed in live third instar larval axons of Control (BG380-Gal4/+; UAS-RAB7:GFP/+) and Khc-73 mutants (BG380-Gal4/+; Khc-73149; UAS-RAB7:GFP/+). Scale bar is 5 μm. (B) Kymographs from live imaging of RAB7:GFP in axons of Control (BG380-Gal4/+; UAS-RAB7:GFP/+) and Khc-73 mutants (BG380-Gal4/+; Khc-73149; UAS-RAB7:GFP/+). (C) Histogram of Retrograde velocities of RAB7:GFP puncta in Control (BG380-Gal4/+; UAS-RAB7:GFP/+) and Khc-73 mutants (BG380-Gal4/+; Khc-73149; UAS-RAB7:GFP/+). n = 5, 8 axons and n = 64, 49 puncta respectively. (D) Histogram of anterograde velocities of RAB7:GFP puncta in Control and Khc-73 mutants. n = 5, 8 axons and n = 49, 27 puncta respectively. (E) LacZ staining in control (Khc73100; pucE69 /+) and Khc-73 mutant (Khc-73149; pucE69 /+) larval motor neuron nuclei in the ventral nerve cord. Scale bar is 10μm. (F) Quantification of mean LacZ fluorescence staining in nuclei for genotypes in (E). N = 279, (6) for control (Khc73100; pucE69 /+) and 535, (11) for Khc-73 mutant (Khc-73149; pucE69 /+), Nuclei, (Ventral Nerve Cords), respectively. (G) LacZ staining in control (Khc73100; pucE69 /+) and Khc-73 mutant (Khc-73149; pucE69 /+) larval motor neuron nuclei in the ventral nerve cord after nerve crush assay. Scale bar is 10μm. (H) Quantification of Anterior and Posterior mean fluorescence of nuclei in (G) expressed as Posterior/Anterior ratio. N = 95, 113, (4) for Khc-73100; pucE69 /+. 219, 300, (10) for Khc-73149; pucE69 /+. Anterior nuclei, posterior nuclei, (Ventral Nerve Cords), respectively. Error Bars are SEM. Student’s t-test. ns-no statistical significance. (TIF) [file pgen.1007184.s008.tif]

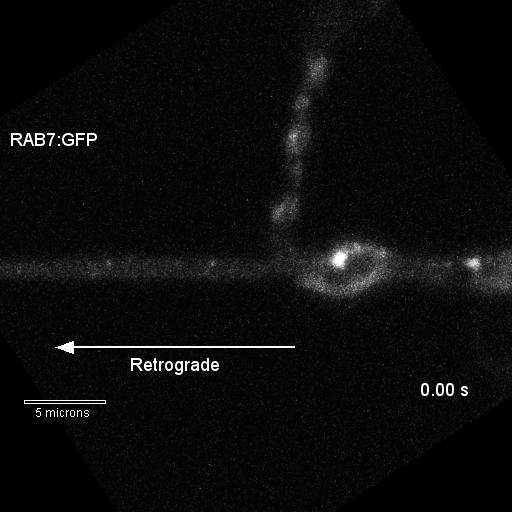

Supplement: S1 Movie — Live imaging of Rab7:GFP in third instar larva, muscle 4 NMJ in control (OK371-GAL4/+; UAS-RAB7:GFP/+). Retrograde direction (toward motoneuron cell body) is to the left. Scale bar is 5 μm. (GIF) [file pgen.1007184.s009.gif]

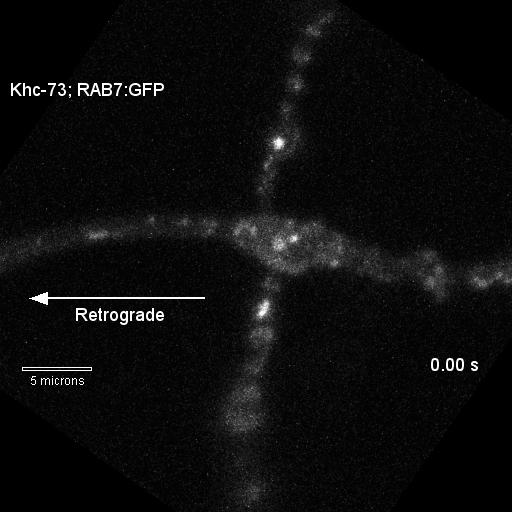

Supplement: S2 Movie — Live imaging of Rab7:GFP in third instar larva, muscle 4 NMJ in Khc-73 mutant (OK371-GAL4, Khc-73149/+, Khc-73149; UAS-RAB7:GFP/+). Retrograde direction (toward motoneuron cell body) is to the left. Scale bar is 5 μm. (GIF) [file pgen.1007184.s010.gif]

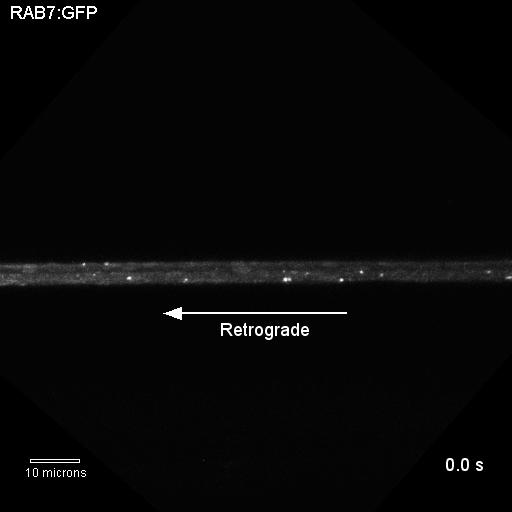

Supplement: S3 Movie — Live imaging of Rab7:GFP in third instar larva axon in control (OK371-GAL4/+; UAS-RAB7:GFP/+). Retrograde direction (toward motoneuron cell body) is to the left. Scale bar is 10 μm. (GIF) [file pgen.1007184.s011.gif]

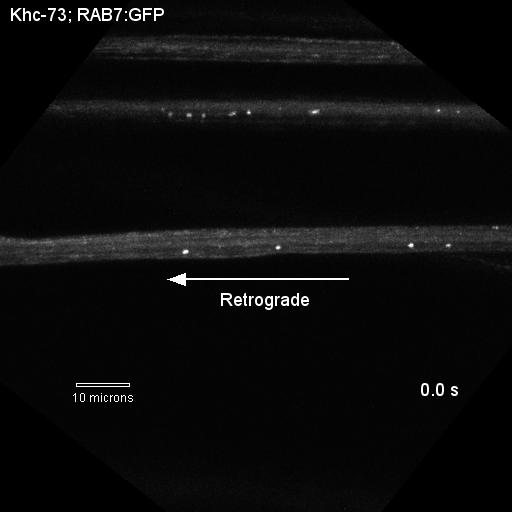

Supplement: S4 Movie — Live imaging of Rab7:GFP in third instar larva axon in Khc-73 mutant (OK371-GAL4, Khc-73149/+, Khc-73149; UAS-RAB7:GFP/+). Retrograde direction (toward motoneuron cell body) is to the left. Scale bar is 10 μm. (GIF) [file pgen.1007184.s012.gif]

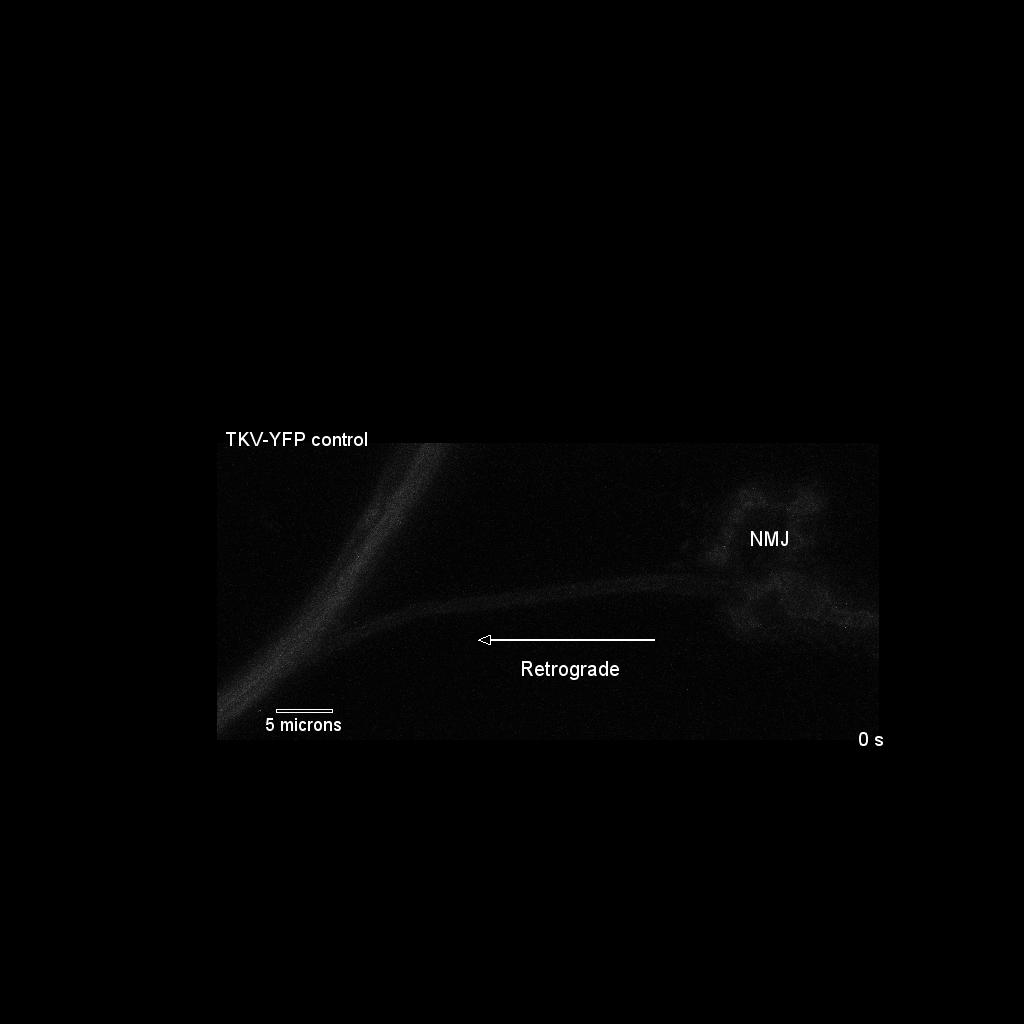

Supplement: S5 Movie — Live imaging of TKV:YFP in third instar larva, muscle 4 NMJ in control (BG380-Gal4/+; OK371-Gal4/+; UAS-TKV-YFP/+). Retrograde direction (toward motoneuron cell body) is to the left. Scale bar is 5 μm. (GIF) [file pgen.1007184.s013.gif]

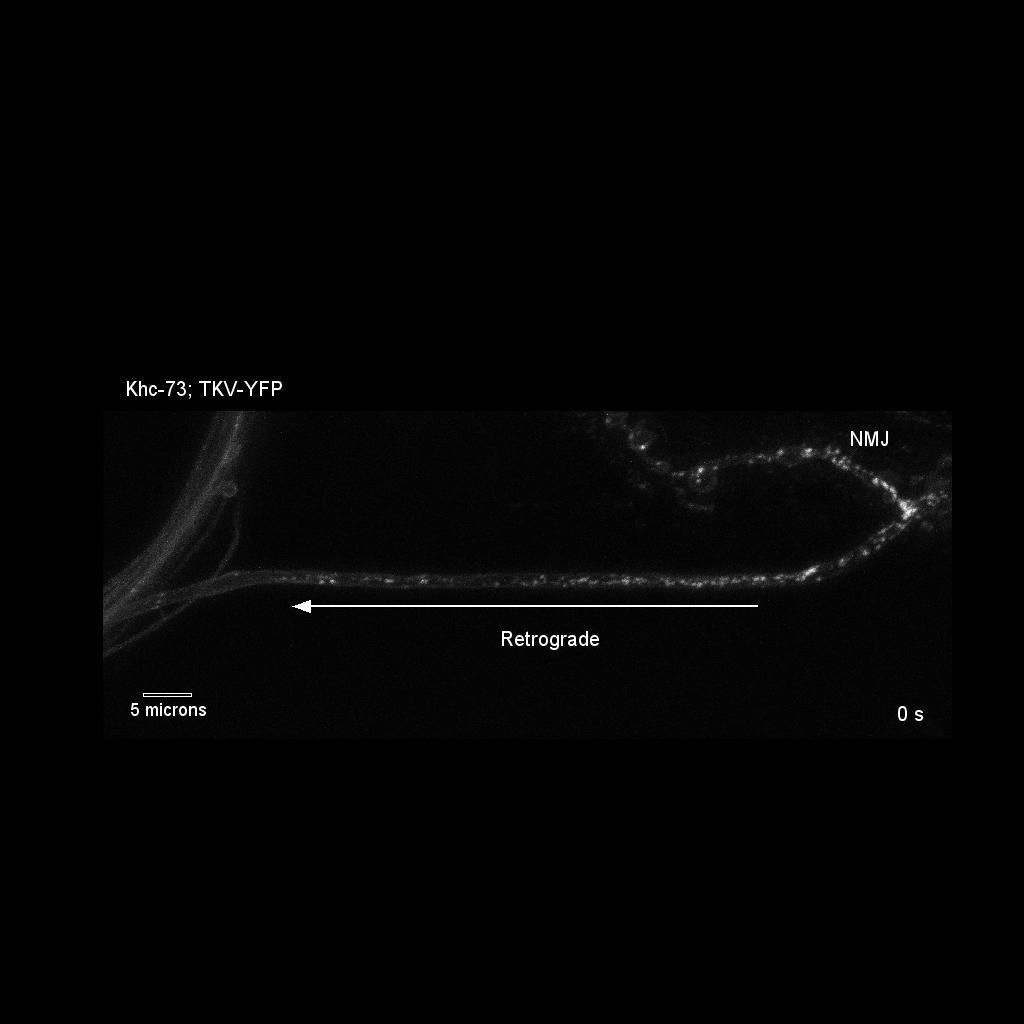

Supplement: S6 Movie — Live imaging of TKV:YFP in third instar larva, muscle 4 NMJ in Khc-73 mutant (BG380-Gal4/+; Khc-73149,OK371-Gal4/ Khc-73149; UAS-TKV-YFP/+). Retrograde direction (toward motoneuron cell body) is to the left. Scale bar is 5 μm. (GIF) [file pgen.1007184.s014.gif]

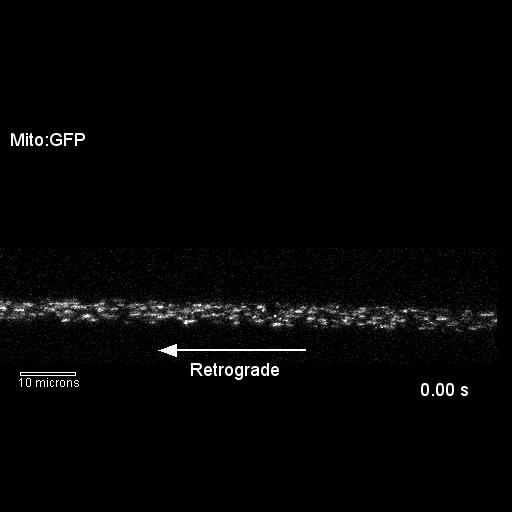

Supplement: S7 Movie — Live imaging of axons in third instar larva of control (OK371-GAL4/UAS-mito:GFP). Retrograde direction is to the left. Scale bar is 10μm. (GIF) [file pgen.1007184.s015.gif]

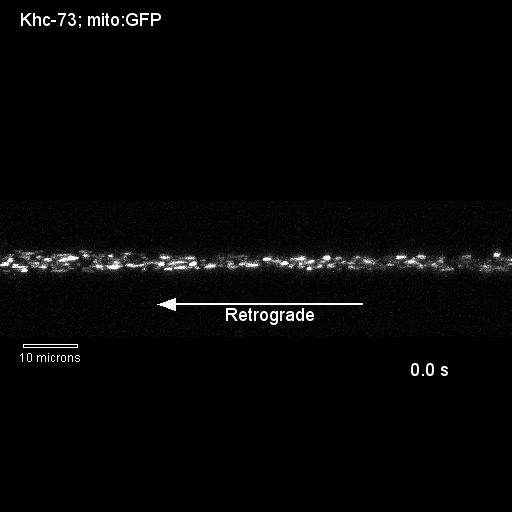

Supplement: S8 Movie — Live imaging of axons in third instar larva of Khc-73 mutant (OK371-GAL4, Khc-73149/UAS-mito:GFP, Khc-73149). Retrograde direction is to the left. Scale bar is 10μm. (GIF) [file pgen.1007184.s016.gif]
